# Multiscale Computational Protocols for Accurate Residue Interactions at the Flexible Insulin–Receptor Interface

**DOI:** 10.1021/acs.jcim.5c00772

**Published:** 2025-05-16

**Authors:** Yevgen P. Yurenko, Anja Muždalo, Michaela Černeková, Adam Pecina, Jan Řezáč, Jindřich Fanfrlík, Lenka Žáková, Jiří Jiráček, Martin Lepšík

**Affiliations:** 1 89220 Institute of Organic Chemistry and Biochemistry of the Czech Academy of Sciences, Flemingovo náměstí 542/2, 166 10 Prague 6, Czech Republic; 2 Department of Physical Chemistry, University of Chemistry and Technology, Technická 5, 166 28 Prague 6, Czech Republic

## Abstract

The quantitative characterization of residue contributions to protein–protein binding across extensive flexible interfaces poses a significant challenge for biophysical computations. It is attributable to the inherent imperfections in the experimental structures themselves, as well as to the lack of reliable computational tools for the evaluation of all types of noncovalent interactions. This study leverages recent advancements in semiempirical quantum-mechanical and implicit solvent approaches embodied in the PM6-D3H4S/COSMO2 method for the development of a hierarchical computational protocols encompassing molecular dynamics, fragmentation, and virtual glycine scan techniques for the investigation of flexible protein–protein interactions. As a model, the binding of insulin to its receptor is selected, a complex and dynamic process that has been extensively studied experimentally. The interaction energies calculated at the PM6-D3H4S/COSMO2 level in ten molecular dynamics snapshots did not correlate with molecular mechanics/generalized Born interaction energies because only the former method is able to describe nonadditive effects. This became evident by the examination of the energetics in small-model dimers featuring all the present types of noncovalent interactions with respect to DFT-D3 calculations. The virtual glycine scan has identified 15 hotspot residues on insulin and 15 on the insulin receptor, and their contributions have been quantified using PM6-D3H4S/COSMO2. The accuracy and credibility of the approach are further supported by the fact that all the insulin hotspots have previously been detected by biochemical and structural methods. The modular nature of the protocol has enabled the formulation of several variants, each tailored to specific accuracy and efficiency requirements. The developed computational strategy is firmly rooted in general biophysical chemistry and is thus offered as a general tool for the quantification of interactions across relevant flexible protein–protein interfaces.

## Graphical abstract

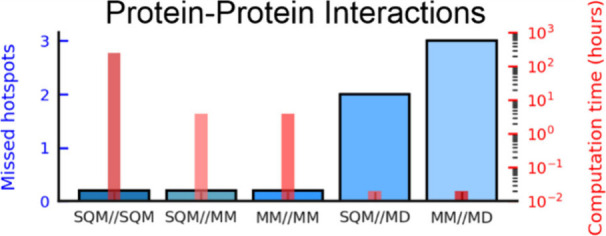

## 1 Introduction

Physics-based computational approaches for the treatment of large and flexible protein–protein interfaces rely primarily on molecular mechanics (MM)-based molecular dynamics (MD) simulations. The calculations of Gibbs free energies can generally be performed using three classes of methods: end point, alchemical or pathway methods.^1–3^ The former class includes the MM/GBSA method, which employs the implicit generalized Born (GB) model to describe the solvation phenomena. When applied to 46 protein–protein complexes, it yielded a satisfactory correlation with the experimental binding affinities.^4^ The more rigorous alchemical transformations between amino acid side chains calculated via thermodynamic integration (TI) or free energy perturbation (FEP) can yield useful estimates of the effects of mutations.^5^ Lastly, various advanced sampling protocols, such as calculation of potential of mean force (PMF) along reaction coordinate, can yield very accurate absolute binding free energies.^6^ However, even these methods are subject to limitations, including insufficient sampling or the inaccuracies of the MM potential.^2^

Conversely, an accurate evaluation of noncovalent interactions necessitates the application of quantum-mechanical (QM) approaches, but these are too demanding for biomolecular systems (reviewed in refs 7–10 ). The utilization of semiempirical QM (SQM) approaches,^7,11^ has emerged as a viable strategy to overcome this impediment. Nevertheless, these methods are characterized by reduced accuracy. Empirical corrections, e.g. for dispersion, hydrogen- or halogen-bonding (H- or X-bonding, respectively)^7,11,12^ have been proposed as a means to address these limitations. Such augmented methods, most notably PM6-D3H4X, provide a highly accurate description of noncovalent interactions in large biomolecular systems, such as protein–ligand complexes for which reliable crystal structures are available.^13^ With respect to this work it is worthwhile to note that the empirical correction for close S···O contacts (PM6-D3H4S)^14^ for the description of chalcogen bonds present in biomolecular complexes^15^ is included in PM6-D3H4X.

To expedite the costly QM calculations in large biomolecular systems, numerous fragmentation schemes, such as fragment molecular orbital (FMO) method or molecular fractionation with conjugate caps (MFCC), have been developed and applied to estimate the binding free energies in protein–ligand complexes (reviewed in ref 16). Another approach is the emerging use of machine-learning QM potentials (reviewed in ref 17). Traditional and successful approaches are QM-cluster and multiscale QM/MM strategies, in which the ligand and the surrounding binding site are treated at the QM level, whereas the remainder is either approximated by the solvent or described using MM, respectively (reviewed in refs 18 and 19). A practical consideration in QM/MM calculations relates to how the QM part is embedded within the MM part. The three possibilities are mechanical, electrostatic, or polarizable embedding, each with their advantages and disadvantages (reviewed in ref 16). Multiscale QM/MM approaches have proven beneficial for modeling protein–ligand interactions by combining accuracy with efficiency. In a recent study, QM/MM-derived charges enhanced the affinity predictions for nine protein targets and 203 ligands.^20^

Calculating free energies of biomolecules requires one to consider the aqueous milieu. In MM-based approaches it is frequently done via explicit models, whereas QM-based approaches mostly utilize implicit models. Previously, implicit COSMO model was reparametrized (COSMO2) and gave a superior performance for model systems as well as protein–ligand complexes.^21,22^ Another approach, which is very efficient is domain decomposition COSMO (ddCOSMO) which achieves near-linear scaling and enables calculations on systems with millions of atoms.^23^

The binding of insulin to its receptor (insulin receptor, IR) produces a protein–protein complex of primary clinical relevance. IR is a receptor tyrosine kinase that plays a crucial role in several biochemical processes in mammalian cells, including glucose homeostasis and protein, carbohydrate, and lipid metabolism. The IR is a homodimeric (αβ/α′β′) glycoprotein composed of extracellular, transmembrane, and intracellular parts. The binding of insulin to the extracellular part (ectodomain) induces conformational changes which are subsequently transduced to the intracellular kinase-signaling domain.^24^ The dysfunction of the IR in humans has been linked to an increased risk of diabetes mellitus or cancer.^25^ The IR ectodomain is composed of multiple domains; the leucine-rich repeat domain (L1), the cysteine-rich region (CR), and the second leucine-rich repeat domain (L2), followed by three fibronectin type III domains (FnIII-1, FnIII-2, FnIII-3). The FnIII-2 domain includes the inset domain whose cleavage separates the α and β chains and generates the α chain C-terminal helix, αCT (Figure 1A). The structural understanding of how insulin engages IR has been growing over the past decade and culminated in a series of cryogenic-electron-microscopy (cryo-EM) structures at a medium resolution of 3–7 Å (Figure 1B).^26–30^ These structures possess up to two insulin binding sites on each IR protomer, namely (i) the primary high-affinity binding Site 1 comprising L1, αCT′ (Site 1a) and FnIII-1′ (Site 1b) IR domains and (ii) secondary low-affinity binding Site 2.^29,30^ Due to the importance and better characterization of the former, this paper focuses only on Site 1.

**Figure 1:**
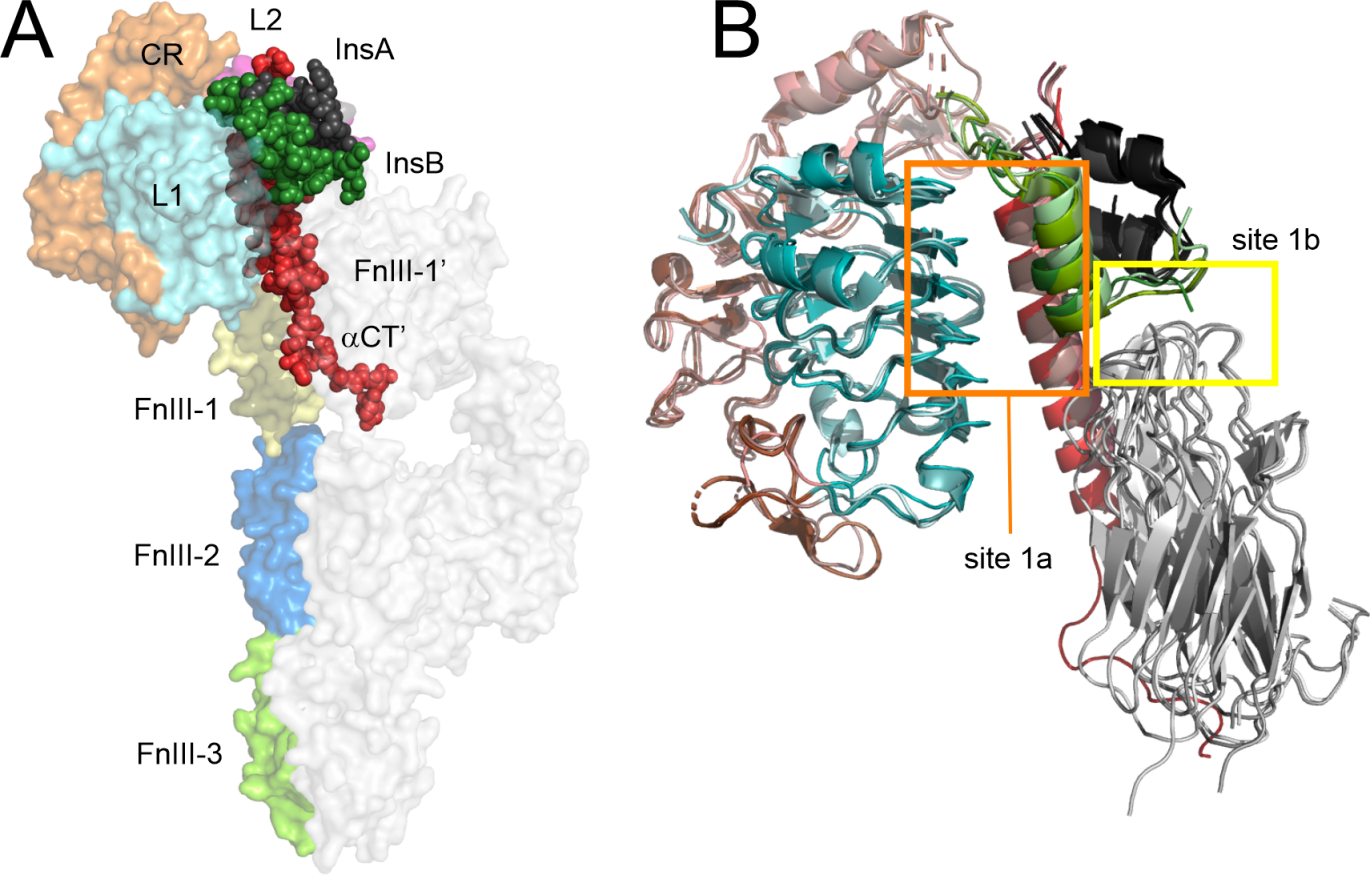
The structures of the insulin–IR complexes with insulin bound to the binding Site 1. **A.** The domain organization of the IR is shown as colored surface in one protomer with the αCT′ helix and the bound insulin shown as spheres (PDB: 6HN5 and 6HN4).^28^ The other IR protomer is shown as white surface. **B.** The conformational plasticity of the IR and insulin as observed in the four cryo-EM structures used in this work for modeling (PDB codes: 6HN5,^28^ 6CE7, 6CE9 and 6CEB,^27^ in different shades). The L2 and FnIII-1 domains are omitted for clarity. Color coding: the L1, CR, L2, FnIII-1, FnIII-2, FnIII-3, and FnIII-1′ domains of the IR are colored cyan, orange, magenta, yellow, light blue, light green, and light gray, respectively. The αCT′ helix and insulin A and B chains are in red, dark gray, and green, respectively. This and the subsequent figures have been prepared in PyMol, ver. 2.0.^31^

The objective of this study is to develop reliable computational protocols for the evaluation of the Gibbs free energy contributions of individual amino acid side chains at large and flexible protein–protein interfaces, as exemplified by the insulin–IR complex. With this tool, we aim to identify the principal noncovalent interactions present at the insulin–IR interface, assess how they depend on conformation and quantify their contributions. From the methodological perspective, we investigate whether the use of the SQM approach (PM6-D3H4S/COSMO2) is advantageous over the use of MM/GB, regarding the accuracy as well as the increased computational time requirements. The results of the developed multiscale protocols are compared with the previous biochemical^32^ and structural^27–30^ experimental data on the insulin–IR binding. This serves to gauge the level of reliability of such computational tools for investigating the molecular details which drive binding at large and flexible protein–protein interfaces.

## 2 Methods

### 2.1 Structures and Modeling

From the dozen of experimental structures of insulin–IR complexes that have become available in the past decade, the cryo-EM complex (PDB: 6HN5)^28^ with the highest resolution of 3.2 Å at the time of calculation initiation was selected to ensure that no unnecessary errors may be introduced in the starting model. The structure was subsequently modified as follows. First, the engineered 33-residue GCN4 leucine zipper was removed. Second, the missing residues of the insulin B chain (B1, B2, B28–B30) as well as IR loops (the residues 160–168, 447–455, and 540–545) were modeled by means of PyMol, ver. 2.0 ^31^ using conformations from other cryo-EM structures (PDB: 6CE7, 6CE9 and 6CEB)^27^ or filled in using the Modeler 9.23 ^33^ program. Subsequently, two loops were remodeled using PyMol.^31^ The loop between Pro160 and Ile174 in the CR domain was adjusted to form a disulfide bridge between Cys169 and Cys188. Next, the loop between the residues 540 and 545 in the IR clashed with the N-terminus of the insulin B-chain. Consequently, this loop and its surrounding residues (Pro537–Pro549) were remodelled to a conformation similar to that observed in the cryo-EM structure (PDB: 6CE7).^27^ Finally, acetyl and *N*-methylamide caps were added using PyMol^31^ to the Lys310 and Tyr675 residues of chain F at the N-termini and the Asn594 residues of chains E, F at the C-terminus, respectively, to avoid the introduction of a non-natural charge. These added caps were far from the insulin-binding site, pointed to the solvent and did not induce any steric clashes. Their relaxation was carried out together with the whole system as detailed below.

### 2.2 Molecular Dynamics Simulations

#### 2.2.1 Setup and Relaxation

The initial minimizations were carried out in AMBER16 ^34^ and the subsequent molecular dynamics (MD) simulations by means of Gromacs 5.1.2 ^35^ using the AMBER ff14SB^36^ force field. The modeled residues in insulin and the loops in the IR (see Section 2.1) were minimized by 5,000 steps of the steepest descent algorithm using the IGB8 implicit solvent model.^37^ Thereafter, the system was solvated with 32,072 OPC3 ^38^ water molecules. Na^+^ and Cl^–^ ions were added to reach the physiological concentration of 0.15 M and neutralize the system. The whole system was then minimized in 2,000 steps using the steepest descent algorithm. This insulin–IR minimized structure without water molecules and ions is made available in public repository.^39^ The cutoff distance for the nonbonded interactions was 14 Å with electrostatics treated with particle–mesh Ewald (PME) approach and the van der Waals interactions with a simple cutoff. Afterward, only the remodeled loops, water and ions were heated, whereas the rest of the protein remained frozen. The sampling of the remodeled loops was enhanced by heating up to 600 K and cooling back to 300 K using the temperature increments of 10 K and the time increments of 50 ps, for the total time of 1 ns. Using a root-mean-square deviation (RMSD) cutoff of 1 Å for structural similarity, two conformational clusters were identified.

#### 2.2.2 MD Runs

From the structure closest to the center of each cluster, four independent equilibration runs of 500 ps were launched in the N*p*T ensemble. The temperature was kept at 300 K using a v-rescale thermostat,^40^ whereas the pressure was maintained at 1 atm using the Berendsen barostat.^41^ Finally, eight independent short production MD runs of 5 ns ensued. Three of these trajectories were selected for further analysis based on the largest number of noncovalent contacts between insulin and the IR (hereafter referred to as traj-1, traj-2 and traj-3; they are available in public repository).^39^ MD snapshots for further interaction energy calculations were obtained by saving frames from traj-1, traj-2 and traj-3 every 15 ps, which yielded ∼ 330 snapshots in each case.

#### 2.2.3 MD Analysis

RMSDs during 5 ns runs of the insulin and the IR backbone were measured. H-bonds were analyzed throughout the 5 ns trajectories and were defined by these structural criteria; the donor (D)···acceptor (A) distance cutoff was set to 3.6 Å, and the D-H···A angle cutoff was set to 180 ± 60°. The nonpolar contacts were analyzed in the last 3 ns of the trajectories, with the threshold for the distance between the non-hydrogen atoms set to 4.1 Å. The CPPTRAJ program of the AMBER16 suite^34^ was used for all the analyses.

### 2.3 System Fragmentation and Interaction Energy Calculations

The complete insulin–IR ectodomain has over 15,000 atoms. For efficient calculations, the system size was reduced by the elimination of the parts of the IR that were distant from the insulin, and surrounding the fragments by the solvent, the so-called QM-cluster approach.^16^ For the assessment of the impact of the IR truncation on the interaction energies, two frames were selected from MD simulations with the greatest number of insulin–IR contacts (referred to as traj-1_maxcont and traj-2_maxcont) and optimized at ff03.r1 protein force field^45^ MM level and IGB = 5 generalized Born (GB) implicit solvent model^46^ (this combination is further referred to as MM/GB). Subsequently, the IR was truncated at increasing distances from the bound insulin (ranging from 3 to 23 Å, with 1 Å intervals) and the truncations capped with acetyl or *N*-methylamide or hydrogen at the N- or C-termini or side chains, respectively. The interaction Gibbs free energies (ΔG_int_) were calculated for these structures by subtracting the Gibbs free energies of insulin and (truncated) IR from the Gibbs free energy of the (truncated) complex (eq 1). The Gibbs free energies were approximated as a sum of the gas-phase energy ΔE at the PM6-D3H4S level,^12,14^ i.e. the PM6 SQM method with empirical corrections for dispersion (D3), hydrogen- (H4) and chalcogen (S) bonding.^11,12,14^ The solvation Gibbs free energy, ΔΔG_solv_, was calculated using COSMO2 implicit solvent model^21^ at the PM6-D3H4S level (eq 2). COSMO2 is the reparametrized version of the original COSMO model with improved performance for model systems as well as protein–ligand complexes.^21,22^ The MOPAC2016 program^42^ with the MOZYME linear-scaling algorithm interfaced to the Cuby4 framework^43,44^ was used.

1 $$\Delta G_{\mathrm{int}}=\Delta G_{\mathrm{cplx}}-\left(\Delta G_{\mathrm{insulin}}+\Delta G_{\mathrm{IR}}\right)$$

2 $$\Delta G=\Delta E+\Delta \Delta G_{\mathrm{solv}}$$

### 2.4 Snapshot Selection, Fragment Optimization, and Interaction Energy Calculations

To reduce the number of MD snapshots for further calculations, the interaction Gibbs free energies were calculated using eq 1 on 990 fragments (330 from each trajectory) of the selected 11 Å size. The Gibbs free energies were approximated using eq 2. To expedite the calculations, no optimization was carried out and the MM level was used. Specifically, the ΔE term was evaluated at the MM/GB level. Ten fragments from each trajectory with the best MM/GB interaction free energies were subjected to different levels of geometry optimization and interaction energy calculations to assess the performance of such protocols.

Three types of geometries were tested: (i) no optimization, i.e. fragmented MD snapshots, (ii) MM/GB optimization and (iii) PM6-D3H4S/COSMO^47^ optimization (the last one only for the ten fragments from traj-1 due to the relatively higher computational requirements). It should be noted that the more accurate COSMO2 method^21,22^ could not be used for optimization as it is only available for single-point energies and thus the original COSMO solvent^47^ was used. The PM6-D3H4S calculations were carried out in two versions - the original and an alternative one without the scaling of charged H-bonds, i.e. with *f*_*charge*_ in eq 1 of ref 12 set to 1, which is more appropriate for the description of such H-bonds in solution. The structures of the fragments are made available in public repository.^39^

Root-mean-square deviations (RMSD) and solvent accessible surface areas (SASA) were analyzed using PyMol, ver 2.0.^31^ The SASAs were obtained by summing the areas of all the accessible grid points around molecules using the default settings.

Interaction energies were calculated using eq 1 for the three types of geometries of 11 Å fragments for the 10 fragments per each trajectory (the exception was that the PM6-D3H4S/COSMO^47^ optimization was carried out for the ten traj-1 fragments only). The Gibbs free energies were approximated using eq 2 at MM/GB and PM6-D3H4S/COSMO2 levels. To describe the combinations of methods, standard computational chemistry notation is used, i.e. vacuum/solvation term of the single-point method//vacuum/solvation term of the optimization method, e.g. PM6-D3H4S/COSMO2//PM6-D3H4S/COSMO (abbreviated as SQM//SQM) or MM/GB//MM/GB (MM//MM).

The PM6-D3H4S/COSMO2 method was selected for its favorable compromise between accuracy and computational cost. It includes empirical corrections for dispersion, hydrogen bonding, and chalcogen interactions, and benefits from improved solvation modeling via COSMO2, but it still inherits some limitations of the PM6 framework such as deviations in geometries compared to DFT.

Moreover, the COSMO and COSMO2 solvation models, being continuum-based, cannot capture discrete solvent effects or specific hydrogen-bonding with solvent molecules. These aspects should be kept in mind when interpreting the calculated interaction free energies.

### 2.5 DFT-D3 Calculations of Characteristic Interaction Motifs

In order to assess the reliability of the PM6-D3H4S/COSMO2 and MM/GB methods for the description of various noncovalent interaction types, the characteristic interaction motifs (amino acid dimers) were identified which corresponded to distinct types of noncovalent interactions (salt bridges, H-bonding, weak H-bonding, chalcogen bonds, and stacking interactions) that were common to the 30 selected snapshots. Three representative structures (fourth snapshot from trajectory 1, designated as tr-1/sn-4, seventh snapshot from trajectory 2, tr-2/sn-7, and fifth snapshot from trajectory 3, tr-3/sn-5) were utilized for a comprehensive analysis.

The dimers were subjected to DFT-D3 geometry optimization employing the B-LYP functional^48,49^ in combination with D3­(BJ)^49,50^ dispersion correction, the def2-TZVPPD^51^ basis set and the COSMO solvation model^47^ with ε_r_ = 78.4 to mimic an aqueous environment. All the DFT geometry optimizations were performed using the TURBOMOLE package, version 7.2.^52^ The interaction energies at this level were compared to those at PM6-D3H4S/COSMO2 and MM/GB levels. In the case of a charged S···O chalcogen bond, the PM6-D3H4S/COSMO, PM6-D3H4/COSMO and MM/GB optimizations were also evaluated. The BLYP-D3 energies were not corrected for basis set superposition error, since the D3 correction is parametrized to cover these effects.^50^

### 2.6 Virtual Glycine Scan (VGS)

The contributions (ΔΔG_int_) of insulin side chains and selected IR residues (within 6 Å of insulin) to the interaction Gibbs free energy ΔG_int_ were calculated by means of a virtual glycine scan (VGS) procedure.^53^ This procedure involves the mutation of all amino acids at the insulin–IR interaction interface (with the exception of prolines and glycines) to glycine, i.e. the replacement of side chains by hydrogen atoms. The ΔΔG_int_ contributions of the residues were calculated as the difference between the original ΔG_int_^wt^ with the wild-type amino acid and the new ΔG_int_^Gly^ with the residue mutated to glycine. The ΔG_int_ values were obtained from the nonoptimized, MM/GB- optimized or PM6-D3H4S/COSMO-optimized 11 Å-fragmented selected MD snapshots (see Section 2.4) as single-point energies at the PM6-D3H4S/COSMO2 and MM/GB levels. The equation for calculating the VGS contributions of individual residues is (eq 3)­

3 $$\Delta \Delta G_{\mathrm{int}}=\Delta {G_{\mathrm{int}}}^{\mathrm{Gly}}-{\Delta G_{\mathrm{int}}}^{\mathrm{wt}}$$

where ΔΔG_int_ is a change in Gibbs free interaction energy upon glycine mutation, computed as the difference between the interaction Gibbs free energy of the wild-type complex (ΔG_int_^wt^) and the complex in which a specific residue is mutated to glycine (ΔG_int_^Gly^).

The binding hotspots, i.e. the residues with significant contributions were identified by compliance to both of these criteria. First, the ΔΔG_int_ value, was ≥ 2 kcal/mol, indicating that the mutation to Gly should result in energetic *destabilization*. The selected threshold (2 kcal/mol) for ΔΔG_int_ was chosen to avoid the potential identification of false positive interactions resulting from intrinsic limitations in the accuracy of the computational methods used. Second, the ΔΔG_int_ were larger than the threshold in all 10 conformations of a particular MD trajectory (traj-1, traj-2 and traj-3). This process made it possible to verify that the interaction in question was dynamically stable, i.e. was preserved in different conformations. The VGS contributions of individual residues were classified as strong (ΔΔG_int_ > 10 kcal/mol), medium (ΔΔG_int_ in range 6–10 kcal/mol) and weak (ΔΔG_int_ in range 2–6 kcal/mol).

### 2.7 Alternative VGS Protocols

To test the effect of the description of charged group H-bonding in the most advanced VGS protocol (SQM//SQM), the H-bond scaling for charged groups was switched off both in geometry optimization and interaction free energy calculation. Due to the higher computational demands, these two VGS setups were applied to ten snapshots from traj-1.

Besides, faster protocols with different geometries and interaction energy calculation methods were tested on all 30 snapshots (i.e., ten snapshots from each from traj-1, traj-2 and traj-3 trajectory). The geometries used were either nonoptimized geometries from MD simulations or MM/GB optimized ones. The interaction energies were evaluated at MM/GB or PM6-D3H4S/COSMO2 levels.

## 3 Results

The results are presented in the following order. First, the local dynamics of the insulin–IR interface are analyzed in terms of noncovalent interactions, specifically H-bonding and nonpolar contacts. Second, the impact of the truncation of the IR on the interaction energies with insulin is investigated, with the aim of determining the optimal size of the insulin–IR model for subsequent SQM (PM6-D3H4S/COSMO) calculations. Next, the geometries of the optimized fragments are compared between the PM6-D3H4S/COSMO andMM/GB levels. As the insulin–IR interaction energies computed at the MM/GB and PM6-D3H4S/COSMO levels do not correlate, the quality of their description of the present noncovalent interactions on smaller model dimers is assessed by comparing them to the reliable DFT-D3/COSMO calculations. Finally, a VGS of amino acid side chains of both, the insulin and the IR at their interface is carried out. Alternative protocols entailing different combinations of methods for geometry optimization and single-point energy calculations are evaluated in terms of both their reliability and efficiency.

### 3.1 Dynamics of the Insulin–IR Interface

Eight short MD runs were carried out on the cryo-EM structure of the insulin–IR complex (PDB: 6HN5) with modeled loops (see Methods, Section 2.1) to capture the local flexibility of the binding interface. At the same time, the objective was to ensure minimal structural variation to facilitate the generation of fragments for the PM6-D3H4S/COSMO calculations with the most similar composition. The three selected trajectories with the greatest number of noncovalent contacts between the insulin and the IR (traj-1, traj-2, and traj-3; available in public repository)^39^ yielded relatively low average root-mean-square deviations (RMSDs) of the IR backbone (2.0 ± 0.6, 1.8 ± 0.3, and 1.8 ± 0.3 Å, respectively) and the insulin backbone (1.0 ± 0.2, 1.0 ± 0.2, and 1.2 ± 0.2 Å, respectively) given the number of flexible loops and termini. Structurally, the largest rearrangements were found at the CR loop (the residues 268–278; 1.0 ± 0.3, 0.7 ± 0.1, 1.0 ± 0.3 Å respectively) and the insulin B-chain C-terminus (the residues B28–B30 which were unresolved in the cryo-EM experiment; 0.4 ± 0.1, 0.4 ± 0.2, and 1.0 ± 0.2 Å respectively).

### 3.2 Hydrogen Bonding

The insulin–IR H-bonds are formed in three pairs of sites (Figure 2A-C): (i) the residues of two helices (helix 1 and helix 2) of the insulin A chain and the IR αCT′ peptide, (ii) the insulin B chain and the cross-linking between Site 1a (L1 domain of IR) and Site 1b (FnIII-1′ loop of IR), and (iii) insulin B chain C-terminus and L1 domain as well as the CR domain loop of IR. We categorize the H-bonds as either well-established or presumable (Table 1) based on the following criteria: the former H-bonds are those with occupancies greater than 70% in each of the three trajectories. The latter group mostly comprise H-bonds which are mediated by residues not resolved in the cryo-EM structure (such as loops in the L1, CR or FnIII-1′ domains of IR or the insulin B chain C-terminus) or are less stable in our short MD simulations (with occupancies below 35% in two of the three trajectories).

**Table 1:** H-Bonding between Insulin and IR from MD

							MD occupancy, %		
Insulin location	Insulin residue	IR	IR residue	Insulin//IR backbone (b) or side chain (s)	In PDB structure	In minimized structure	traj-1	traj-2	traj-3
Well-established H-bonds									
A-chain helix 1	Val A3	αCT′	Asn 711	b//s	yes	yes	87.5	87.2	94.3
A-chain helix 2	Asn A18		Arg 717	b//b	yes	yes	99.8	99.5	99.9
B-chain helix	Ser B9	L1	Arg 65	s//s	yes	yes	96.9	95.5	96.6
	Glu B13			s//s	no	no	79.5	70.3	94.5
B-chain C-terminus	Phe B24		Asn 15	b//s	no	yes	99.6	99.7	99.5
Presumable H-bonds									
A-chain helix 1	Glu A4	αCT′	Asn 711	s//s	no	no	87.2	84.8	36.4
A-chain helix 2	Asn A21		Arg 717	b//b	no	yes	97.9	35.6	94.6
B-chain N-terminus	Asn B3	FnIII-1′	Lys 544^a^	s//s	no	yes	42.2	40.3	no
				s//b	no	no	78.6	38.9	15.8
B-chain α helix	His B10		Ser 540^a^	s//b	no	no	91.3	76.5	no
B-chain	Glu B21	L1	Lys 40	s//s	yes	yes	71.0	32.2	26.7
C-terminus									
B-chain	Thr B30	CR	Gln 272	b//b	no	yes	84.2	96.6	11.1
C-terminus			Gly 273	b//b	no	no	90.0	96.4	10.1

a Residues missing in the 6HN5 cryo-EM structure.

**Figure 2:**
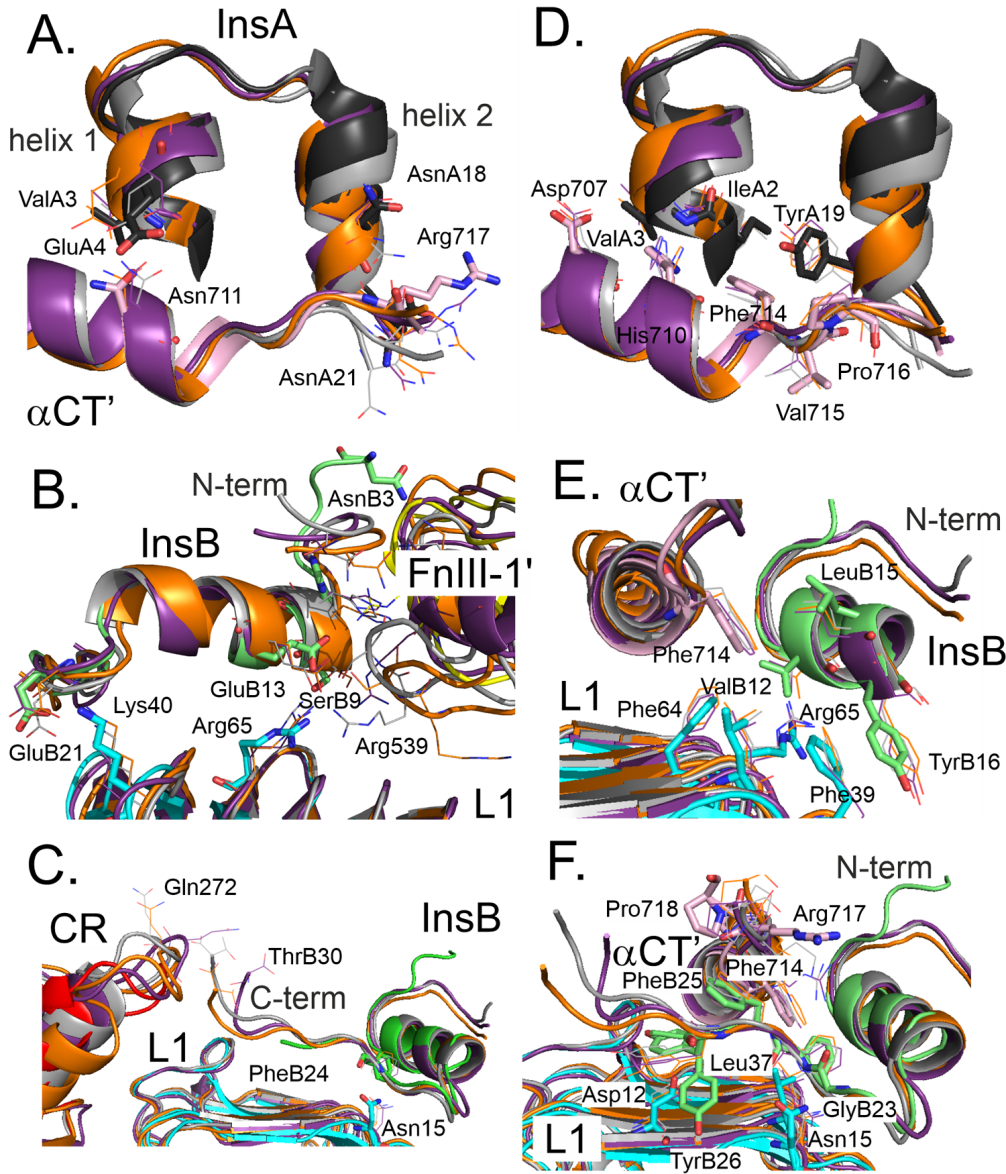
Regions with H-bonding (**A-C**) and nonpolar contacts (**D-F**) between insulin and IR. The cryo-EM structure cartoon is colored by IR domains (L1 cyan, CR red, FnIII-1′ yellow, αCT′ pink) and insulin chains (A chain black, B chain magenta). The cartoons of three snapshots from the three trajectories are shown in gray (traj-1), orange (traj-2) and violet (traj-3). Important residues are shown in sticks for the cryo-EM structure and as lines for the three snapshots. **A.** αCT′/insulin chain A, **B.** L1 and FnIII-1′ domains/insulin chain B (αCT′ omitted for clarity), **C.** L1 and CR domains/insulin chain B, **D.** αCT′/insulin chain A, **E.** L1 domain and αCT′/insulin chain B (its C-terminal residues B17–B30 omitted for clarity), **F.** L1 domain and αCT′/insulin chain B.

Our computational approach has augmented the information provided by the cryo-EM structure. Besides H-bonds present in the cryo-EM structure and maintained at approximately 90% of the MD frames (Val A3···Asn 711; Asn A18···Arg 717; Ser B9···Arg 65), there are also well-established H-bond pairs absent from the cryo-EM structure which are formed only upon minimization or short MD (Phe B24···Asn 15; Glu B13···Arg 65; Table 1; Figure 2 A-C). On the other hand, several presumable H-bonds (Glu A4···Asn 711 and Asn A21···Arg 717) are a result of local structural changes during MD within the αCT′ IR domain and of the change of mutual orientation of the insulin A chain with respect to the αCT′ domain of the IR. Another presumable H-bond, the Glu B21...Lys 40 pair, is located at the solvent-exposed interface between the L1 domain and the insulin B chain and is thus present at low occupancies (Table 1; Figure 2 B). The transient nature of the H-bonding of Thr B30 with Gln 272 and Gly 273 (with the occupancy of 10% in traj-3) attests to the flexibility of the insulin B-chain C-terminus.

### 3.3 Non-Polar Contacts

Other stabilizing interactions between insulin and IR are nonpolar contacts (Table 2). They occur at similar regions (Figure 2 D-F) as H-bonding: (i) the residues of two helices (helix 1 and helix 2) of insulin A chain and the IR αCT′ peptide, (ii) the insulin B chain type II β-turn (B8) and central α-helix (B12, B16) with L1 domain and αCT′ peptide and (iii) insulin B chain C-terminus (B23–B26) and IR L1 domain and the αCT′ peptide.

**Table 2:** Non-Polar Contacts between Insulin and IR Residue Pairs from MD^a^

				Number of pairwise atom–atom contacts			Occupancy per contact during MD trajectories, %			
Insulin location	Insulin residue	IR	IR residue	traj-1	traj-2	traj-3	traj-1	traj-2	traj-3	average
Region 1										
A chain helix 1	Ile A2	αCT′	Phe 714	1	7	7	90.0	50.0	62.9	67.6 (m)
A chain helix 1	Ile A2	αCT′	His 710	1	2	4	90.0	90.0	55.0	78.3 (s)
A chain helix 1	Val A3	αCT′	Asp 707	5	7	7	94.0	77.1	78.6	83.2 (s)
A chain	Tyr A19	αCT′	Phe 714	3	4	6	86.7	92.5	63.3	80.8 (s)
C-terminus										
A chain	Tyr A19	αCT′	Val 715	5	2	5	70.0	100.0	70.0	80.0 (s)
C-terminus										
A chain	Tyr A19	αCT′	Pro 716	15	11	13	45.3	62.7	60.0	56.0 (m)
C-terminus										
Region 2										
B chain type II β-turn	Gly B8	αCT′	Glu 706	1	1	3	100.0	100.0	83.3	94.4 (s)
B chain helix	Val B12	L1	Leu 37	1	1	1	90.0	100.0	90.0	93.3 (s)
B chain helix	Val B12	L1	Phe 64	2	2	3	55.0	55.0	36.7	48.9 (w)
B chain helix	Val B12	L1	Arg 65	2	3	4	80.0	63.3	60.0	67.8 (m)
B chain helix	Val B12	αCT′	Phe 714	2	2	4	60.0	70.0	37.5	55.8 (m)
B chain helix	Leu B15	αCT′	Phe 714	6	7	6	93.3	84.3	91.7	89.8 (s)
B chain helix	Tyr B16	L1	Phe 39	25	33	19	13.7	25.2	13.1	17.3 (w)
Region 3										
B chain type I	Gly B23	L1	Asn 15	3	3	3	100.0	93.3	96.7	96.7 (s)
β-turn										
B chain	Phe B24	L1	Leu 37	5	5	4	86.0	88.0	2.5	58.8 (m)
C-terminus										
B chain	Phe B24	αCT′	Phe 714	7	8	3	58.6	76.3	66.7	67.2 (m)
C-terminus										
B chain	Phe B25	αCT′	Pro 716	4	1	4	60.0	100.0	62.5	74.2 (s)
C-terminus										
B chain	Phe B25	αCT′	Arg 717	11	17	20	84.5	55.3	65.0	68.3 (m)
C-terminus										
B chain	Phe B25	αCT′	Pro 718	6	2	4	76.7	95.0	80.0	83.9 (s)
C-terminus										
B chain	Tyr B26	L1	Asp 12	7	7	5	74.3	64.3	88.0	75.5 (s)
C-terminus										

a The contacts are classified into three categories as weak (w, average of the occupancies per contact over trajectories <50%), medium (m, average 50-70%), and strong (s, average ≥ 70%).

Nonpolar contacts are classified as strong, medium and weak based on the trajectory averages of total occupancies per contact over three MD trajectories (Table 2). Most of the pairwise nonpolar contacts were C–H···π weak H-bond or π···π stacking between large residues: Tyr A19···Pro 716, Tyr B16···Phe 39, and Phe B25···Arg 717 (Figure 2 D-F). There was also a hydrophobic cluster comprising residues Val B12, Phe 714, Leu 37, Phe 64 connecting insulin B chain to αCT′ peptide and L1 domain (Figure 2 E), thus stabilizing the insulin–IR binding interface.

### 3.4 The Selection of the Truncated IR Model Size

The insulin–IR complex consists of 15,514 atoms, which precludes efficient calculations by use of the PM6-D3H4S/COSMO method. The IR molecule was thus truncated at increasing distances from the bound insulin from 3 to 23 Å in two MD snapshots from traj-1 and traj-2, which corresponds to system sizes ranging from 1,431 to 7,914 atoms. The fragmentation is accompanied by variations in the total charges from −5 to +6. In these insulin–IR fragments, we calculate PM6-D3H4S/COSMO2 interaction energies. The interaction energy profiles for the two snapshots share similar features (Figure S1). The largest 23 Å fragments are taken as reference and smaller fragments are compared to them, both, in terms of interaction energies and total charges. The 17-Å fragments have the closest values but are still too large for efficient calculations (5,652 and 5,541 atoms for the two selected snapshots). A yet smaller model fragmented at 11-Å distance has a reasonable size (∼3,500 atoms), capturinging 96% and 91%, respectively, of the interaction energies of the 23-Å sized fragments. Structurally, these models contain most of residues from the IR binding site (L1, CR, L2, FnIII-1′, and αCT′ domains; compare the whole insulin–IR minimized structure with the fragments in the public repository).^39^

### 3.5 The Geometries of Optimized Truncated Models

Using this fragment size of 11 Å, ten snapshots from traj-1 were selected according to the highest MM/GB interaction energy (Figure S2). The effect of the geometry-optimization method (MM/GB or PM6-D3H4S/COSMO) was examined on these ten snapshots by comparing to the nonoptimized MD geometries (see the fragments in the public repository).^39^ Not surprisingly, MM/GB optimized geometries were more similar to the nonoptimized MD than the PM6-D3H4S/COSMO optimized geometries were (0.48–0.64 Å vs 0.99–1.70 Å, respectively; Tab. S1). This can be attributed to the similarity of MM potentials (note the explicit/implicit solvent difference), in contrast to the QM potential. The latter, however, should lead to higher-quality description of geometries of noncovalently bound complexes, especially when quantum effects are present.^12^

The solvent-accessible surface areas (SASA) of the optimized snapshots indicate that PM6-D3H4S/COSMO optimization leads to slightly higher overall compactness of the insulin–IR models (the SASA values decreased by ∼ 10% with respect to their MM/GB counterparts, Table S2). Moreover, the size of the insulin expressed as the distance between the C_α_ atoms of Phe B1 and Lys B29, is significantly reduced in PM6-D3H4S/COSMO optimized complexes (by up to ∼ 9% with respect to the nonoptimized MD geometries). On the contrary, no effect of insulin–IR size reduction has been observed in the case of the MM/GB optimization protocol.

### 3.6 Interaction Energies in 11-Å Fragments

Interaction Gibbs free energies at MM/GB and PM6-D3H4S/COSMO2 levels were calculated for ten snapshots from traj-1 in nonoptimized, MM/GB-optimized and PM6-D3H3S/COSMO-optimized MD geometries (Table S3 and the fragments in the public repository).^39^ The method of geometry optimization had a major impact on the resulting interaction Gibbs free energies. Going from non-optimized via MM/GB-optimized to PM6-D3H4S/COSMO-optimized, there were shifts toward higher stabilization. The average interaction Gibbs free energies for these three types of geometries were −59.9, −84.6, and −248.7 kcal/mol, respectively. The higher interaction energies in the last case can be attributed to the increased compactness of these structures (see above). This compactness is caused by stronger H-bonds and salt bridges, evidenced by shorter distances, as well as by additional stabilizing S···O chalcogen bonds, which can only be properly described by the PM6-D3H4S/COSMO2 method (see below).

This shift in interaction Gibbs free energies exhibited a systematic trend; the MM/GB energies calculated at nonoptimized and MM/GB-optimized geometries in 30 snapshots (see the fragments in the public repository)^39^ had the coefficient of determination R^2^ of 0.58 and PM6-D3H4S/COSMO2 energies calculated for MM/GB-optimized and PM6-D3H4S/COSMO-optimized geometries (ten snapshots) had the coefficient of determination R^2^ of 0.42. In contrast, there was no correlation between the interaction Gibbs free energies calculated with MM/GB or PM6-D3H4S/COSMO on their respective geometries (Figure S3). Altogether, these findings quantify the dependence of the resulting interaction free energies on geometry optimization as well as single-point interaction energy calculation methods.

### 3.7 Interaction Energies in Small Model Dimers

This lack of correlation between MM//MM and SQM//SQM interaction free energies in 11Å-fragmented models prompted us to compare the quality of the description of the dominant types of the present noncovalent interactions on small model dimers optimized at the reliable DFT-D3/COSMO level. First, the nature and relative strength of four types of interactions (neutral and charged hydrogen bonds, π···π stacking and chalcogen bonds) was visualized (Figures S4 and S5). Second, ranking of the interaction energies by the three methods was evaluated on dimers extracted from three MD snapshots (see Methods, section 2.5). Both, MM/GB and PM6-D3H4S/COSMO2 methods rank all the noncovalent interactions in the same order as DFT-D3/COSMO (Table S4), yielding a perfect correlation. However, the root mean-square error (RMSE) was much higher for MM/GB (6.5 kcal/mol) than for PM6-D3H4S/COSMO2 (1.2 kcal/mol). The qualitative difference was observed for the charged S···O chalcogen bond which was assessed as repulsive by MM/GB, but as mildly attractive for PM6-D3H4S/COSMO2 and DFT-D3/COSMO. The reason for the failure of MM/GB is that the S and O atoms are both partially negatively charged (−0.13 and −0.82 *e*, respectively) and the energy minimum structure separates them to more than 5 Å (Figure S6). It is worth noting that PM6-D3H4S/COSMO optimization without the S···O correction leads to an artificial shortening of the S···O contacts and only PM6-D3H4S/COSMO remedies the situation and renders the optimized geometry very similar to that of DFT-D3 (Figure S6). Therefore, the PM6-D3H4S/COSMO2 method should be preferred for both, geometry optimization and interaction free energy calculations when describing charged S···O chalcogen bonding. For larger 11-Å models, we expect the superiority of the PM6-D3H4S/COSMO2 model over MM/GB due to the nonadditivity effects on top of the pairwise noncovalent interactions tested in this section. The DFT-D3 optimization of dimers was carried out using 16 CPUs, and the computational cost varied significantly depending on the initial structure and presence of charged S···O interactions. The wall times ranged from several hours to up to 2 days (approximately 40–336 CPU-hours), especially for dimers that included slowly converging chalcogen-bonded systems. For this reason, we limited DFT-D3 calculations to a representative subset of three snapshots.

### 3.8 Identification of Interaction Hotspots at the Insulin–IR Interface by Means of a Virtual Glycine Scan

The Gibbs free energy contributions of amino acid side chains at the insulin–IR interface were calculated at the SQM//SQM level of theory (PM6-D3H4S/COSMO2 single-point energies on PM6-D3H4S/COSMO-optimized (geometries) using the VGS procedure. In the case of the insulin, the number of residues was 46 (all the residues excluding glycines and prolines). For the IR, all the residues within 6 Å from the insulin were considered (i.e., about 50 residues, depending on the snapshot).

Fifteen insulin residues whose side chains significantly contributed to the interaction with the IR (hereafter called hotspots) were identified by a PM6/D3H4S-based VGS (Table 3A). Based on their Gibbs free energy contributions, these residues were classified as strong, medium or weak (for the criteria, see Section 2.6). Most of the hotspots bind in Site 1a. Only Asn B3, Gln B4 and His B10 interact with the FnIII-1′ domain of the IR and thus belong to Site 1b. The Glu B13 and Ser B9 residues have been calculated to be the strongest contributors to the insulin–IR interactions at the junction of Site 1a (Arg 65 of the L1 domain) and Site 1b (Arg 539 of FnIII-1′) of IR (Figure 3A). On the other side of Site 1b, the Asn B3 and Gln B4 residues strongly interact with the flexible loop of residues 540–544 of FnIII-1′ (Figure 3B). At the center of Site 1a, the well-known Phe B24 insulin residue has a calculated contribution of 9 kcal/mol, which is attributable to an H-bond with Asn 15 in the L1 domain and nonpolar contacts with Leu 37 and Phe 714 (Figure 3C, Table 3A). Insulin hotspots have mostly been located on the B-chain α-helix (B9, B10, B12, B13, B15, B16) and the B chain C-terminus (B24, B25) of the insulin. Remarkably, 14 of the 15 hotspots were implicated in H-bonding and/or nonpolar contacts as found in MD simulations (cf. sections 3.2 and 3.3). A semiquantitative comparison of the interaction Gibbs free energies of PM6-D3H4S/COSMO2 with the occupancies from MD for these residues has revealed that there is only some agreement. Because of the superior quality of the description of the noncovalent interactions of the former method,^13^ the PM6-D3H4S/COSMO2 results should be considered more reliable. The only insulin hotspot found in VGS but not in MD is Gln B4.

**Table 3:** Interaction Hotspots Calculated by VGS at the PM6-D3H4S/COSMO2//PM6-D3H4S/COSMO Level Are Ordered by Decreasing ΔΔG_int_ Values,^a^ with Intramolecular Interactions Also Shown

**A.** Insulin residues				
Strength	Insulin residues^a^	ΔΔG_int_ Mean (std. dev.)	IR partner (IR domain)	Intramolecular Interactions
Strong	Glu B13^a,b^	29.09 (7.87)	Arg 65 (L1), Arg 539, Ser 540 (FnIII-1′)	Leu B17
	Ser B9^a^	18.20 (6.14)	Arg 65 (L1)	Cys B7, Val B12, Glu B13
	Asn B3^b^	14.83 (4.85)	Ser 540, Asn 541, Lys 544 (FnIII-1′)	Glu B13, Ala B14
	Glu A4^a^	14.03 (2.06)	Asn 711 (αCT′)	Gly A1, Thr A8
	Gln B4^b^	12.11 (4.78)	Trp 493, Lys 544 (FnIII-1′)	Val B2
	Tyr B16^a^	12.04 (4.29)	Phe 39, Lys 40 (L1)	Val B12, Gly B20
	Phe B25^a^	11.19 (1.73)	Pro 718, Asn 711 (αCT′)	Tyr A19, Thr B27
	His B10^b^	10.40 (2.93)	Ser 540, Lys 544 (FnIII-1′)	Gln B4, Ala B14
	Val B12^a^	10.15 (3.05)	Leu 37, Phe 39, Arg 65 (L1)	Gly B8, Ser B9, Leu B15, Tyr B16
Medium	Phe B24^a^	9.28 (2.82)	Arg 14, Asn 15 (L1), Leu 37 (L1), Phe 714 (αCT′)	Leu B15
	Glu B21^a^	7.23 (2.60)	Lys 40 (L1)	-
	Val A3^a^	6.48 (2.23)	Asp 707, Asn 711 (αCT′)	Cys A7, Thr A8
	Ile A2^a^	6.43 (1.73)	Asn 711, Phe 714 (αCT)	Cys A6, Gln *A*5, Leu B15, Leu B11
	Tyr A19^a^	6.05 (2.03)	Phe 714 (αCT′)	Gly A1, Leu A16, Phe B25
Weak	Leu B15^a^	4.42 (1.17)	Phe 714 (αCT′)	Cys B19, Val B18, Leu B11

**B.** IR residues			
Strength	IR residues	ΔΔG_int_ Mean (std. dev.)	Insulin partner residues
Strong	Arg 65 (L1)	27.95 (7.79)	Ser B9, Glu B13
	Asn 711 (αCT′)	25.41 (1.83)	Ile A2, Val A3, Glu A4
	Arg 717 (αCT′)	17.86 (3.82)	Asn A21, Phe B25
	Lys 40 (L1)	17.43 (3.51)	Glu B21
	Arg 498 (FnIII-1′)	15.97 (2.69)	Cys B7
	Ser 540 (FnIII-1′)	15.85 (2.66)	Asn B3, His B10
	Arg 14 (L1)	14.46 (2.81)	Phe B24
	Phe 714 (αCT′)	11.55 (0.65)	Tyr A19, Phe B24
	Lys 544 (FnIII-1′)	11.25 (7.37)	Asn B3
	Arg 271 (CR)	10.99 (3.66)	Lys B29
	Asp 12 (L1)	10.53 (1.87)	Tyr B26
Medium	Asn 15 (L1)	8.29 (3.93)	Gly B23, Phe B24
	Phe 39 (L1)	6.86 (1.14)	Tyr B16, Val B12
Weak	Asp 496 (FnIII-1′)	5.98 (2.11)	Cys B7
	Val 715 (αCT′)	4.76 (1.36)	Tyr B26, Thr B27

a In kcal/mol, averaged over ten MD snapshots from traj-1 trajectory.

b Note: a and b superscripts denote Site 1a and Site 1b, respectively.

**Figure 3:**
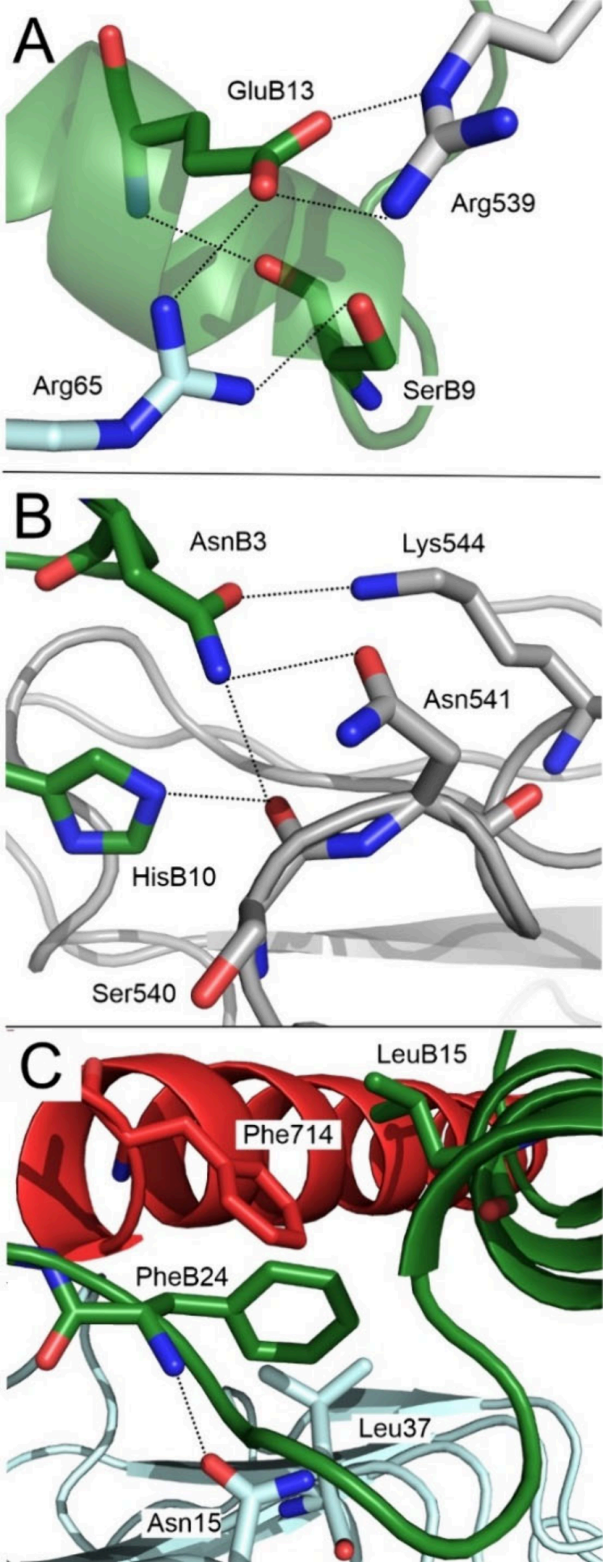
Selected interactions at the insulin–IR interface from PM6-D3H4S/COSMO-optimized MD snapshots with high Gibbs free energy contributions in VGS. **A.** Site 1*a*/1b junction **B.** Distant part of Site 1b, **C.** Site 1a. H-bonds are shown as dotted lines.

The reason for this minor discrepancy is the flexible nature of some interactions which are described differently by these methods. For example, Gln B4 of the flexible insulin B chain N-terminus forms two H-bonds with Leu 538 and Ser 540 in the flexible loop of the FnIII-1′ domain in the PM6-D3H4S/COSMO-optimized structures but it fails to do so in the MD trajectories.

The VGSs of the IR residues interacting with insulin yielded 15 hotspots (Table 3B). These residues were mostly situated within the L1, the αCT′, and the FnIII-1′ domains. They were classified as strong (11 residues), medium (two residues) and weak (two residues) contributors (for the criteria, see Section 2.6). Most of these hotspots (only except Gln B4 in the insulin and Arg 14, Arg 271, Arg 496, and Arg 498 in IR) were identified in MD simulations as participating in H-bonding and/or nonpolar contacts (Figure 3 and Sections 3.2, 3.3). Those not identified in MD were, for example, located on flexible loops (Arg 271 within the CR domain; Asp 496 and Arg 498 within the FnIII-1′ domain).

Considering the calculated ΔΔG_int_ for all the residues, it is possible to estimate the energetic significance of Site 1a versus Site 1b binding. The separation of Site 1a and Site 1b residues VGS contributions to total insulin–IR interaction energy ΔG_int_ shows that the IR Site 1b, constitutes ∼ 25% of the total stability of the insulin–IR complex (Table S5).

### 3.9 Ranking of Interaction Hotspots Using Alternative Virtual Glycine Scan Protocols

There are multiple variants of the most rigorous VGS protocol (PM6-D3H4S/COSMO2 interaction free energies at PM6-D3H4S/COSMO geometries) with different free energy contributions as well as timings. The first option was to switch off the upscaling of charged interactions in the PM6-D3H4S method for both, geometry optimization and interaction energy calculation. Energy differences of 4–7 kcal/mol were found for the charged Glu B13, Glu B21, and Lys B29 and for the uncharged residue Asn B3 interacting with the charged Lys 544 (Figure 4; Tab. S6). The reasons are in part caused by different geometries; in the case of B13, its interaction partners change from Arg 65 and Arg 539 when the scaling is on to Tyr 67 and Asn 527 when the scaling is off. Another cause may be sought in the different energetics. The effect on uncharged interactions was unsurprisingly small (1–2 kcal/mol).

**Figure 4:**
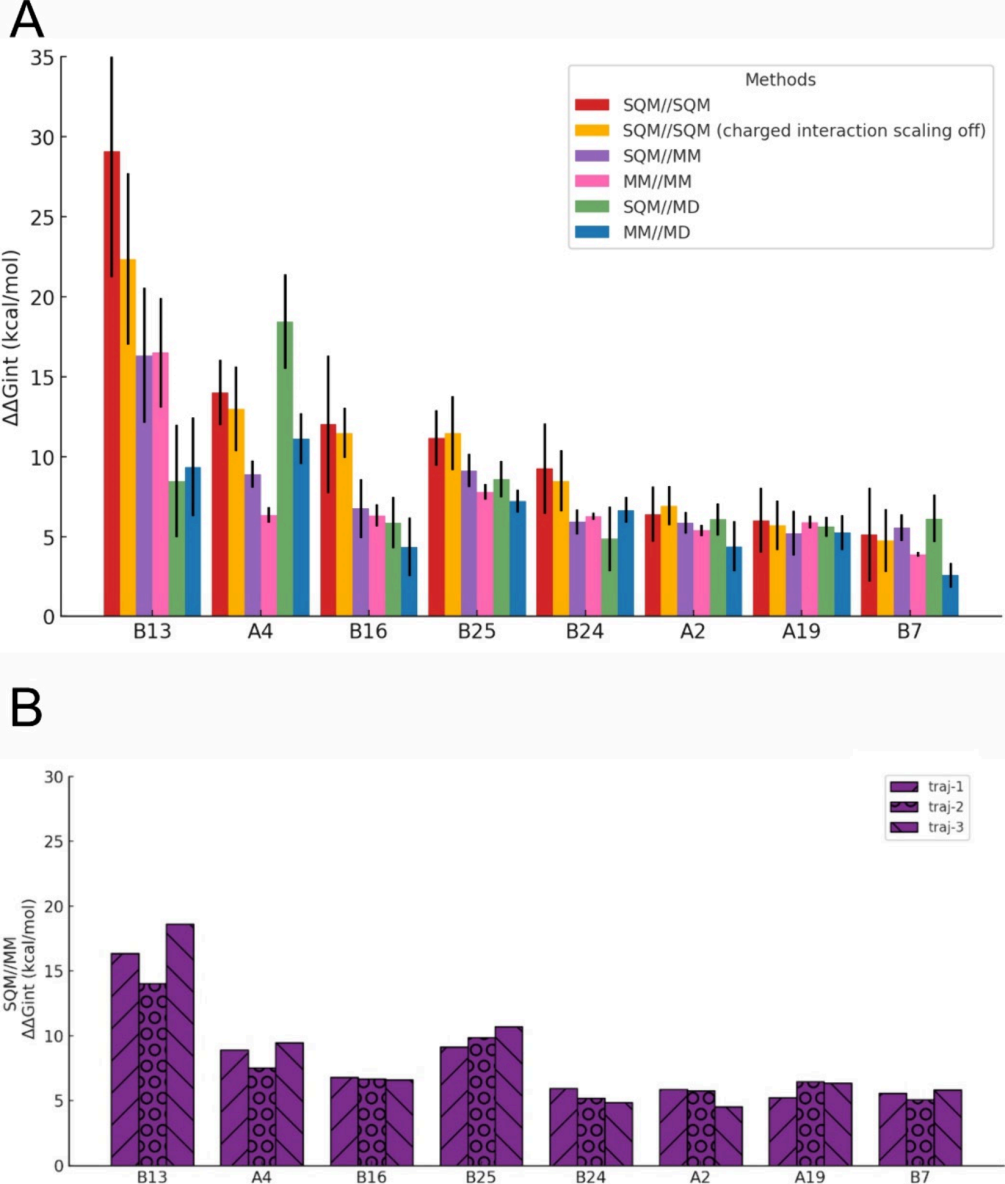
VGS energy contributions, ΔΔG_int_ (kcal/mol), for eight selected insulin residues. **A.** Comparison of various computational protocols for ten snapshots from traj-1, SQM//SQM stands for PM6-D3H4S/COSMO2//PM6-D3H4S/COSMO, MM//MM stands for MM/GB//MM/GB. **B.** Comparison of SQM//MM values for ten snapshots from each, traj-1, traj-2 and traj-3.

The other VGS options (MM/GB or no optimization and MM/GB interaction energies) were faster and thus could be extended to all 30 snapshots from the three independent MD trajectories. Overall, they identified most of the insulin hotspots found by the original VGS protocol but their overall energetic ordering differed (Figure 4; Figure S7, Tab. S6–S10). Generally, this is because MM/GB and PM6-D3H4S/COSMO2 VGS energy contributions were, in some cases, rather different from each other even when evaluated on the same set of geometries (i.e., the same optimization protocol). Specifically, the greatest discrepancies were observed for charged residues, especially Glu B13, which is involved in two salt bridges with Arg 65 (L1 domain) and Arg 539 (FnIII-1′ domain). The MM/GB approach yielded lower values for electrostatic interactions.

The comparison of various VGS protocols revealed trends in Gibbs interaction free energy contributions (ΔΔG_int_) decreasing (in the absolute values) in the order SQM//SQM ≫ MM//MM ≫ MM//MD (Figure 4A). The correlation between PM6-D3H4S/COSMO2 and MM/GB energetic contributions for MM/GB-optimized and nonoptimized MD geometries was quite satisfactory, with Pearson correlation coefficients, *r*, of 0.83 and 0.82, respectively. However, there was a qualitative difference for a small subset of Gibbs free energy contributions which were considered as destabilizing by PM6-D3H4S/COSMO2 but stabilizing by MM/GB. For MM/GB-optimized geometries, it was about 5% of the data points, whereas for MD geometries, the percentage increased to roughly 10% (Figure S8, green rectangles). This trend suggested that MM/GB optimization decreases the error of MM/GB energetics, while it remains high for MM//MD.

Comparing the original SQM//SQM VGS protocol with the alternative ones for their ability to identify key hotspots, it was found that the SQM//MM and MM//MM protocols were able to identify all of them but SQM//MD missed two (B3 and B4) and MM//MD missed three hotspots (B3, B4, and B15) (Table S11).

The comparison of several VGS protocols suggests that in the cases where ranking of hotspot residues is important, higher-level methods for geometry optimization and single-point energy calculations are needed. For instance, while both SQM//SQM and SQM//MM protocols identified the strongest (B13) and the weakest (B5) residues correctly, they differed in the ranking of the five strongest interactions. The SQM//SQM ranking (Table 3A) lists B13, B9, B3, A4, and B4, whereas MM//MM (Table S6) ranks B13, B25, A4, B12, and B10. These protocols also differ in classifying weak (1 vs 7), medium (5 vs 8), and strong (9 vs 1) interaction hotspots.

As can be seen from the variability of the free energy contributions (Figure 4) it is advisable to utilize multiple trajectories and several snapshots. The energetics of noncovalent interactions and thus also VGS results are sensitive to the conformations of the insulin–IR complex.

Regarding the computational cost of the SQM//SQM VGS protocol for these large systems (11-Å IR fragment of ∼ 3,500 atoms), it is approximately 80 times higher than that of MM//MM (Table S11). Thus, if only identification of hotspots is requested without special care about their ordering, SQM//MM protocol may be the most practical. On the other hand, MM//MD protocol is the fastest but should rather be avoided due to its tendency to significantly underestimate interaction energies. The choice of protocol should thus balance accuracy with computational efficiency, depending on the specific requirements of the study.

## 4 Discussion

### 4.1 Augmented Description of Protein–Protein Interfaces by Means of Computational Approaches

Cryo-EM is ideally suited to study large protein–protein complexes and as such has been used repeatedly to help elucidate the structural changes within insulin receptor (IR) domains induced by insulin binding, reviewed in ref 54. By comparing these static structures, researchers were able to propose several mechanisms of the transition of IR to its active conformation.^55,56^ However, the medium resolution of current cryo-EM maps (typically 3–6 Å), together with the inability to resolve flexible loop regions leaves room for improvements, e.g. via integrative modeling.^57,58^ In case of insulin–IR complex, molecular dynamics (MD) was utilized to investigate temporal stability of inter-residue contacts of insulin in four sites on IR (Site 1 and 2 on each IR protomer).^29^ Here, we use only short MD starting from the remodeled cryo-EM structure to locally sample conformations of insulin–IR complex. The need to retain the general architecture of the binding interface for further SQM treatment prevented us from carrying out more thorough conformational sampling. Also, the number of trajectories (three) and snapshots (ten from each trajectory) is limited and was chosen as a compromise between the representativeness of the ensemble of structures and the computational cost needed for different variants of VGS.

The credibility of this comprehensive approach including consideration of multiple conformations has been proven by the agreement of the identified hotspots to the experimental data available in the literature.^32^ We are aware that the exact parameters of the setup defined here for the insulin–IR complex (11-Å-sized IR fragment, 3 trajectories and 10 snapshots each) cannot be generalized and may need to be tested for each protein–protein system studied in the future. The findings of this study suggest that a short MD is necessary to capture the local dynamics of the binding region and that the MM/GB approach is suited for a quick optimization of MD snapshots. The evaluation of the interaction Gibbs free energies using the PM6-D3H4S/COSMO2 method is based on physicochemical principles and should hold in any protein–protein system. However, it is recommended to be careful when dealing with charged interactions, which are inherently difficult to describe accurately.

### 4.2 Semiempirical Quantum Mechanical Methods for Protein–Protein Interactions

The SQM methods with corrections for noncovalent interactions used here (PM6- D3H4S) represent the state-of-the-art for biomolecular applications and have proven (in the PM6-D3H4X version which additionally includes halogen-bonding correction denoted as X) that they can deliver DFT-quality results on protein–ligand crystal structures in affordable time.^13^ The previous pioneering use of a PM6-D3H4/COSMO version for protein–protein binding starting from a well-resolved crystal structure and using VGS identified residue side chains critical for binding.^53^ In this work, we leave the area of crystallographically well-defined interactions and start off from medium-resolution cryo-EM structure, model the missing loops and generate conformations using MD. Tens of snapshot are selected hierarchically using MM/GB method and then different levels of optimization and single-point energies are tested. The recent state-of-the-art SQM method, PM6-D3H4S/COSMO2, including corrections for S···O contacts is used as the highest level of optimization. To increase the efficiency, 11-Å sized fragments of IR were used, similarly to previous works on different proteins.^53,59^

A reliable assessment of binding energetics in protein complexes requires a quantitative description of all types of noncovalent interactions. A benchmark study evaluating the performance of various SQM methods and DFT with respect to the reference CCSD­(T) energies was carried out for protein–ligand complexes.^22^ The PM6-D3H4X method performed the best from the SQM category with the error of 9.4% being close to DFT-D3 (7.2%).^22^

Nonetheless, the PM6-D3H4S/COSMO2 approach should be interpreted with caution in cases where exact geometrical parameters or subtle electronic rearrangements are critical. Despite empirical corrections, semiempirical QM methods are inherently approximate, and their applicability may vary depending on the nature of the molecular system under investigation. Future work may incorporate explicit water molecules or hybrid QM/SQM schemes to refine specific solvent effects where needed.

Here, we compare the fast MM/GB method with the latest PM6-D3H4S/COSMO2 method and DFT-D3/COSMO. Their ranking of interactions is small amino acid dimers is nearly perfect. The only qualitative difference is seen for the short S···O contacts, so-called chalcogen bonds. These occur both in protein–ligand and protein–protein complexes^15,60,61^ and belong to a large class of σ-hole interactions of QM origin.^60–62^ MM/GB fails to describe the geometries and energetics of σ-hole interactions due to electrostatic repulsion, except explicit corrections via extra points.^63,64^

### 4.3 Multiscale Approaches for Protein–Protein Interactions

Molecular dynamics (MD) simulations, combined with free energy estimation methods such as molecular mechanics/generalized Born surface area (MM/GBSA)^65^ and molecular mechanics/Poisson–Boltzmann surface area (MM/PBSA)^66^ are extensively used to investigate protein–protein interactions. These approaches provide dynamic insights into binding energetics but depend on force fields that do not capture electronic effects like polarization or charge transfer. Advanced sampling techniques, including metadynamics and adaptive sampling, have been developed for the physics-based refinement of protein–protein complexes in contact map space.^67^

Hybrid quantum mechanics/molecular mechanics (QM/MM) methods enhance accuracy by applying QM calculations to specific interaction regions while treating the remainder of the system with MM. These approaches have been successfully applied to study the modulation of protein–protein binding by protein residue methylation.^68^ However, QM/MM approaches are computationally intensive and thus are rarely applied to large protein–protein complexes, particularly those involving highly flexible interfaces.

Machine learning (ML) models including deep learning and graph-based approaches, have emerged as valuable tools for predicting binding affinities and mutation effects in protein–protein interactions.^69,70^ These models leverage extensive structural and sequence databases but often struggle with novel, highly flexible protein interfaces lacking sufficient experimental annotations. Despite their potential, ML approaches may lack interpretability and require substantial experimental data for training, making them less reliable for mechanistic studies of new or highly dynamic protein–protein interaction systems.

Our study bridges the gap between MM-based methods and QM/MM approaches by employing semiempirical quantum mechanical (SQM) calculations (e.g., PM6-D3H4S/COSMO2) combined with MD-derived snapshots, virtual glycine scanning (VGS), and fragmentation techniques. Unlike traditional MM approaches, this protocol captures nonadditive effects such as cooperative hydrogen bonding and chalcogen interactions, which are critical in protein–protein complexes. Compared to QM/MM methods, our approach is significantly more computationally efficient while still providing enhanced accuracy over MM-based free energy calculations. In addition, it can provide accurate residue contributions to the hormone-receptor binding energy.

### 4.4 VGS Limitations, Insights, and Protocols

Prior to the analysis of the VGS results, it is worthwhile to enumerate the inherent limitations: (i) only side chain energetic contributions are determined (ii) glycine and proline residues are omitted; (iii) changes of conformation due to mutation are neglected (i.e., after mutation, there is no optimization); (iv) interactions of three and more residues are not described properly. Despite these limitations, VGS can give interesting quantitative insights.

Qualitatively, the VGS results align well with the occupancies of H-bonds and nonpolar contacts (see Tables 1 and 2) as well as with the experimental alanine scanning mutagenesis^32^ data and recent cryo-EM^24,28–30,72^ studies (Table S12). Quantitatively, VGS gives ranking of residues by the strength of noncovalent interactions. The experimental affinities of insulin mutated to alanine at positions B13, A2 and A19 are very low relative to the wild-type insulin,^32^ which is supported by our computational data (the ΔΔG_int_ for these amino acid units being high, see Table 3A). The two phenylalanine residues (B24 and B25) at the C-terminus of insulin B chain, with crucial roles in IR binding^54^ have significant ΔΔG_int_ values (Table 3A). On the other hand, the remaining B chain C-terminal residues, B26–B30, in most cases,^54^ do not provide a substantial contribution to interaction energy between insulin and IR partners as follows from structural analysis of MM/GB optimized snapshots extracted from three MD trajectories. This can be explained by the detachment of B26–B30 from the B chain hydrophobic core during the binding of the insulin to the IR and less pronounced interactions with IR. This energetic feature is supported by experimental data, which indicate that insulin, lacking the terminal amino acids B27–B30 is equipotent to natural insulin.^32^ However, B26 position has ambiguous properties because its modifications especially in B27–B30-shortened and B26-amidated insulins, can result in “superpotent” insulins.^73–75^

Regarding the efficiency of the VGS protocol, we note that the benchmark protocol (SQM//SQM) can only be used for a limited number of snapshots if the highest accuracy is required. Changing the optimization protocol to MM/GB renders the calculations 2 orders of magnitude faster at a cost of different relative ordering of the residue contributions. Based on the low interaction energies, it is not recommended to take MD snapshots directly without optimization. Using the experience gained in our recent protein–ligand study,^13^ future VGS studies should use the SQM//MM setup in order to reduce the computational cost substantially and in the same time the energetic ranking could be close to the SQM//SQM treatment.

## 5 Conclusions

A hierarchical computational protocol has been developed that involves molecular dynamics, fragmentation, and a virtual glycine scan to investigate the binding interactions between insulin and its receptor. Its distinctive attribute is the exploitation of recent advancements in semiempirical quantum mechanical and implicit solvation models, which make up the PM6-D3H4S/COSMO2 method. This approach yields interaction Gibbs free energies that are qualitatively superior to those obtained by the molecular mechanics/generalized Born (MM/GB) method. The reason is that only the former method can describe nonadditive effects present in noncovalent interactions across the insulin-receptor interface. Fifteen hotspot residues on insulin and 15 on the insulin receptor were captured by use of virtual glycine scan and their ranking determined by PM6-D3H4S/COSMO2. They were perfectly in line with the available biochemical and structural data, which demonstrates the accuracy of the method. Faster variants of the protocol have been identified which are less accurate but can serve for a quick identification of interaction hotspot residues. Besides energetics, these computational protocols give atomistic insights into interactions across highly flexible protein–protein interface and thus can be used to design specific insulin mimetics with higher affinity to the receptor. As the approach is physics-based, it is not system-specific and can thus be used for the quantification of interactions across other relevant flexible protein–protein interfaces.

## Supplementary Material



## Data Availability

The following molecular structures produced in this work are available at Mendeley repository:^39^ insulin–IR minimized structure without water molecules and ions (.pdb); MD files: starting structures (.pdb) and binary trajectories (.xtc); fragments of insulin–IR complexes from three MD trajectories optimized by various methods (.pdb); and DFT-optimized amino acid dimers from three MD trajectories (.xyz)

## Notes

**Figure S1**: Dependence of interaction Gibbs free energy ΔG_int_ on system size (fragmentation distance) for two MD snapshots. **Figure S2**: MM/GB interaction Gibbs free energies for insulin–IR across 990 MD snapshots from three trajectories. **Figure S3**: Correlation plot between MM//MM and SQM//SQM protocols for ten traj-1 snapshots. **Figure S4**: Structural analysis of key insulin–IR residue pairs (e.g., H-bonding, salt bridges, π-π stacking). **Figure S5**: NCI analysis of key insulin–IR residue pairs (e.g., H-bonding, salt bridges, π-π stacking). **Figure S6**: Structural depiction of chalcogen bonding at the IR CR/L2 domain using four computational methods. **Figure S7**: VGS contributions across protocols **Figure S8**: Correlation plots between SQM and MM VGS results. **Table S1**: RMSDs for PM6-D3H4S/COSMO and MM/GB optimized geometries. **Table S2**: Solvent-accessible surface areas and insulin compactness metrics for 10 selected snapshots. **Table S3**: Interaction energies for all snapshots (three trajectories) with various optimization schemes. **Table S4**: Energies for selected dimers computed by DFT-D3, SQM, and MM/GB. **Table S5**: Site 1b contribution to total ΔG_int_. **Table S6**: Residue-wise VGS ΔΔG_int_ values for different protocols. **Table S7**: Lists of insulin hotspots based on optimized geometries. **Table S8**: Lists of IR hotspots based on optimized geometries. **Table S9**: Hotspots on insulin from nonoptimized MD geometries. **Table S10**: Hotspots on IR from nonoptimized MD geometries. **Table S11**: Timing and performance comparisons of all tested protocols. **Table S12**: Comparison of VGS results with experimental alanine scanning mutagenesis (PDF)
