# Supplementary material for: Multiscale Computational Protocols for Accurate Residue Interactions at the Flexible Insulin–Receptor Interface
Source: J Chem Inf Model. 2025 May 16;65(11):5690–705. doi: 10.1021/acs.jcim.5c00772 (PMC12152935; doi:10.1021/acs.jcim.5c00772)
Supplement: Supplementary file 1 [file ci5c00772_si_001.pdf]

SUPPLEMENTARY INFORMATION

Multiscale Computational Protocols for Accurate Residue Interactions at the Flexible Insulin–Receptor Interface

Yevgen P. Yurenko,[1] Anja Muždalo,[1] Michaela Černeková,[1, 2] Adam Pecina,[1] Jan Řezáč,[1] Jindřich Fanfrlík,[1] Lenka Žáková,[1] Jiří Jiráček,[1] Martin Lepšík[1*]

[1] Institute of Organic Chemistry and Biochemistry of the Czech Academy of Sciences, Flemingovo náměstí 542/2, 166 10, Prague 6, Czech Republic

[2] Department of Physical Chemistry, University of Chemistry and Technology, Technická 5, 166 28, Prague 6, Czech Republic

* Corresponding author: M. Lepšík (lepsik@uochb.cas.cz), ORCID: 0000-0003-2607-8132

Supplementary Figures

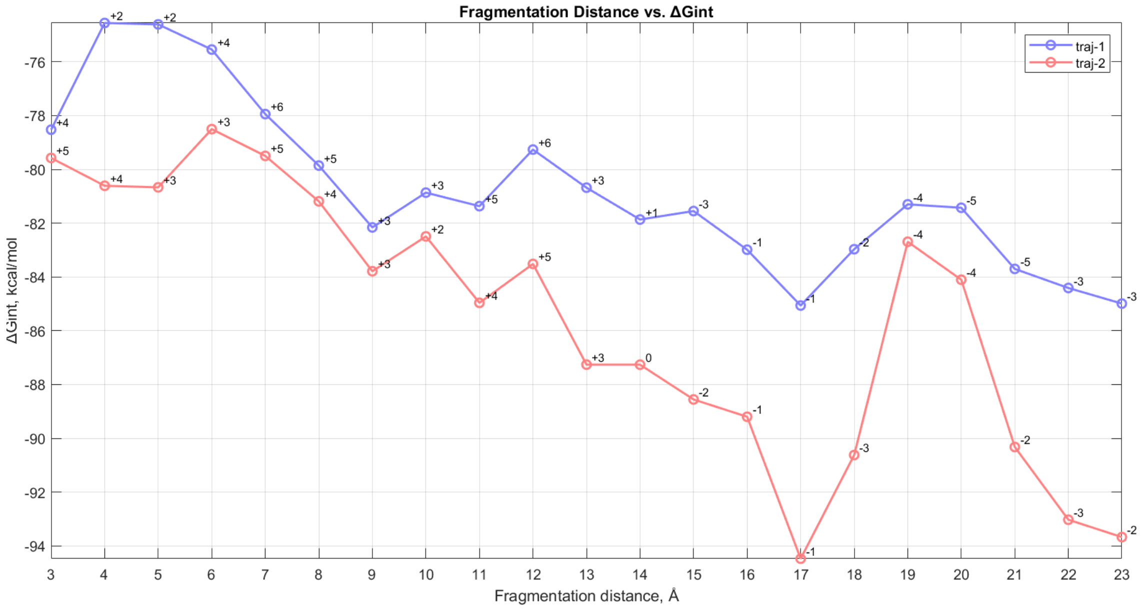

Fig. S1. The dependence of interaction Gibbs free energy ΔG_{int} (in kcal/mol) between insulin and IR and size of the system calculated at the PM6-D3H4S/COSMO2 level. The x-axis shows the fragmentation distance (3-23 Å surroundings from insulin). The data are shown for two MD snapshots: traj-1_maxcont (blue) and traj-2_maxcont (red). The total charge of each insulin–IR fragment is indicated.

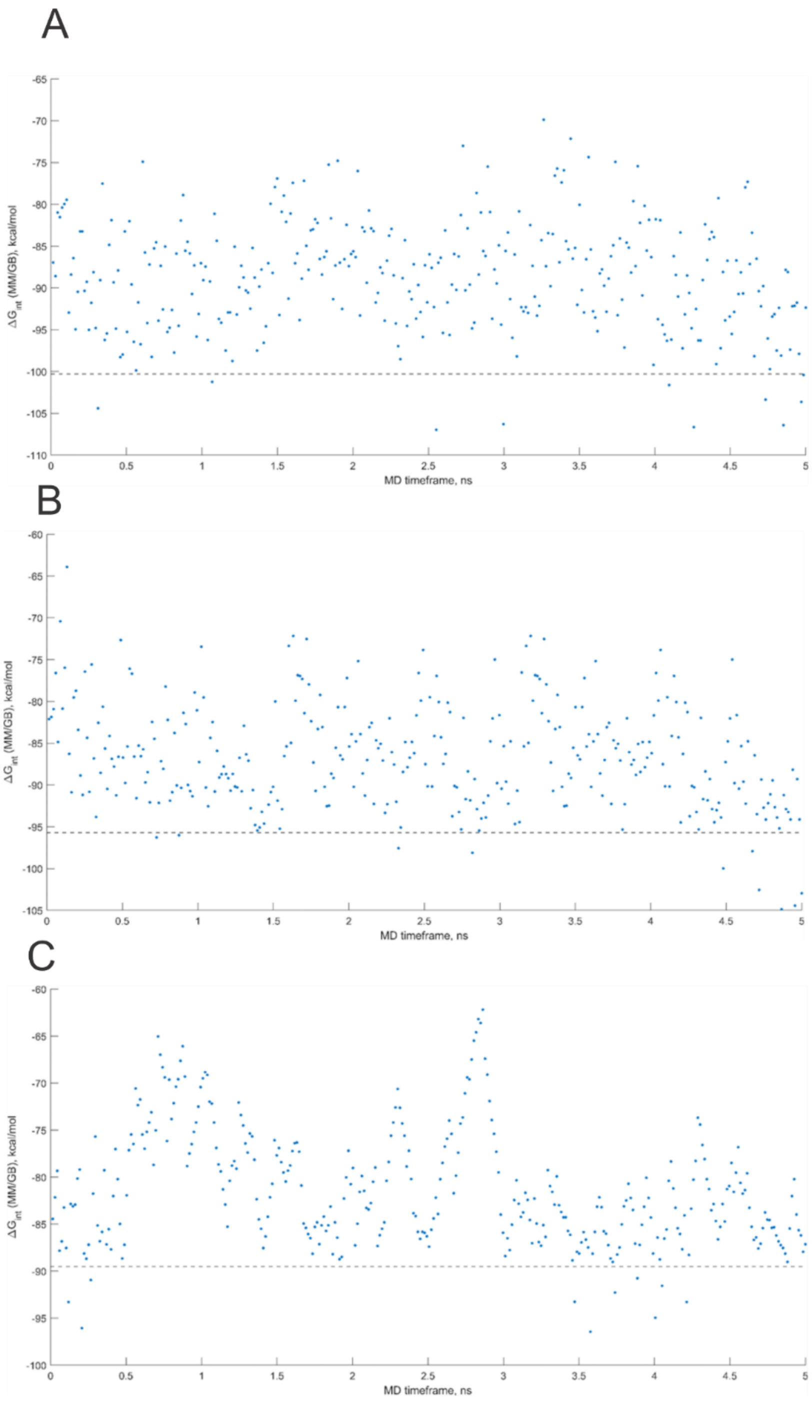

Fig. S2. Molecular mechanics (MM)/generalized Born (GB) implicit solvent interaction Gibbs free energies (in kcal/mol) between insulin and IR for 330 MD snapshots from each of the trajectory **A.** traj_1, **B.** traj_2, **C.** traj_3. The dashed horizontal line separates the ten selected snapshots per each trajectory with the greatest magnitudes of ΔG_{int}.

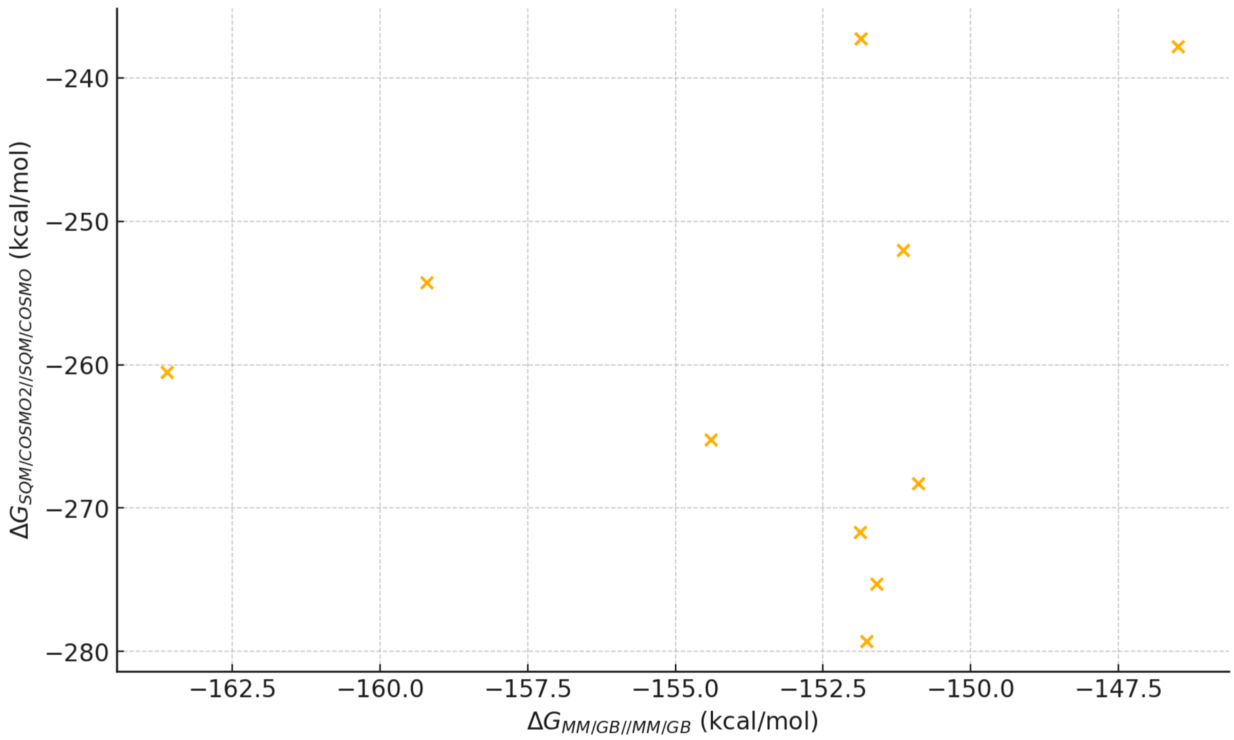

Fig S3. The lack of correlation between insulin–insulin receptor (IR) Gibbs interaction free energies for 10 snapshots of the traj-1 trajectory calculated using the MM//MM and SQM//SQM protocols.

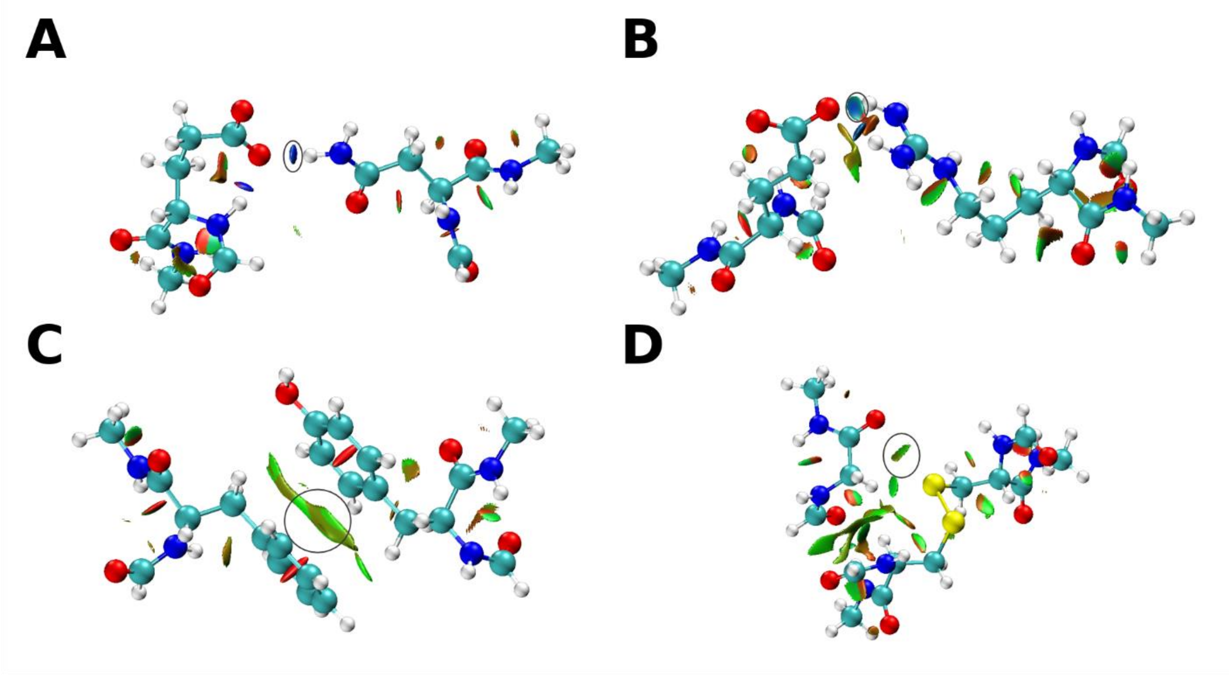

Figure S4. Model dimers and their non-covalent interaction isosurfaces[1] (A) Glu A4 (insulin)…Asn 711 (αCT', IR) H-bonding depicted as blue isosurface indicative of strong attraction. (B) Glu B13 (insulin)…Arg 65 (L1, IR) salt bridge characterized by a light blue, yellow isosurface corresponding to strong electrostatic stabilization. (C) Tyr B16 (insulin)…Phe 39 (L1, IR) π…π stacking shown as diffuse green isosurfaces representing weak dispersion interactions. (D) Gly 463…Cys 468 (L2, IR) chalcogen bond interaction in green. The isodensity surfaces that correspond to the described types interactions are marked by circles.

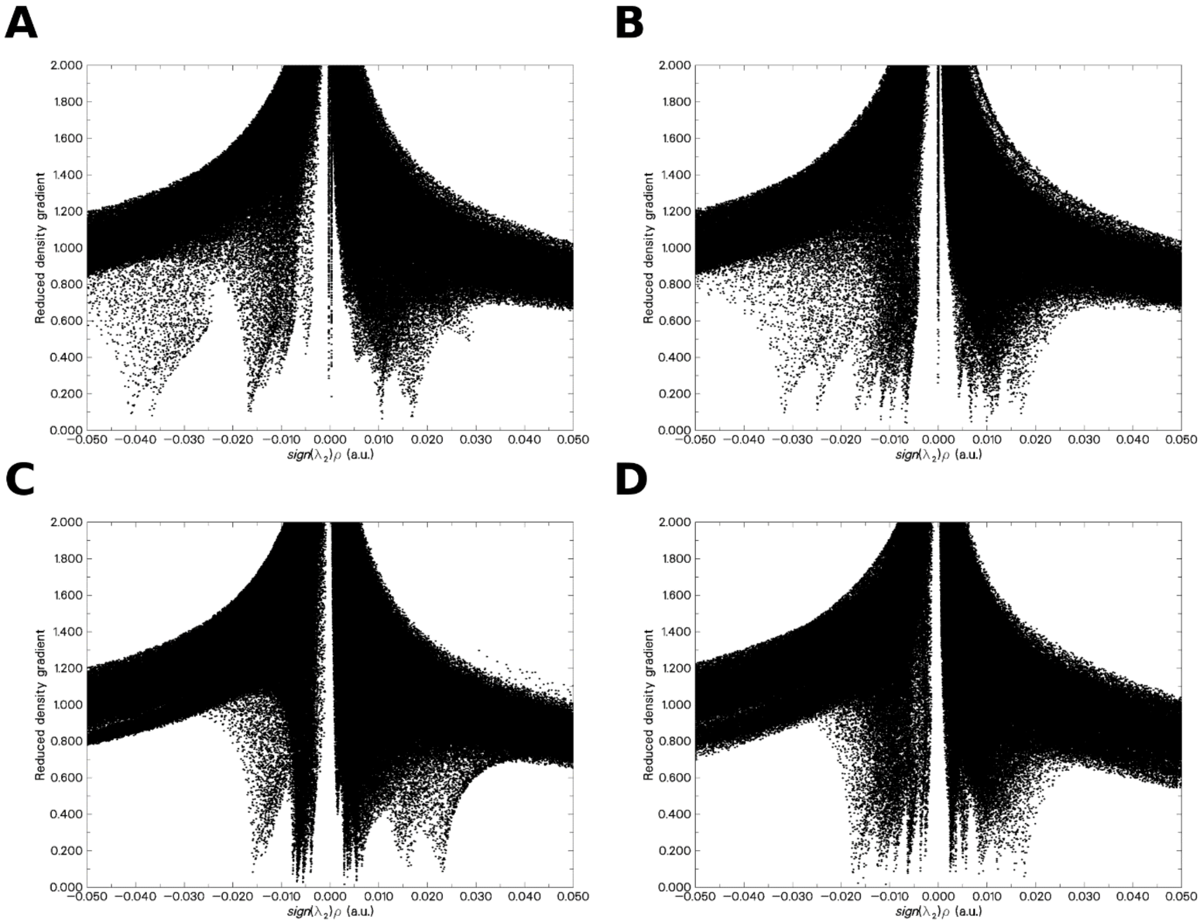

Figure S5 Non-covalent interaction scatter plots[1] for the amino acid pairs from Fig. S4. The negative regions of sign(λ_2)ρ correspond to attractive interactions, while positive regions indicate repulsive forces**.** (A) Glu A4 (insulin)…Asn 711 (αCT', IR) H-bonding with a strong attractive region (sign(λ_2)ρ ≈ −0.02 to −0.04 a.u.). (B) Glu B13 (insulin)…Arg 65 (L1, IR) salt bridge showing the most intense attractive interaction (sign(λ_2)ρ ≈ −0.04 to −0.05 a.u.). (C) Tyr B16 (insulin)…Phe 39 (L1, IR) π…π stacking characterized by weak dispersion forces (sign(λ_2)ρ ≈ −0.01 to 0.01 a.u.). (D) Gly 463…Cys 468 (L2, IR) chalcogen bond interaction displaying moderate attraction due to S…O interaction (sign(λ_2)ρ ≈ −0.02 to −0.03 a.u.).

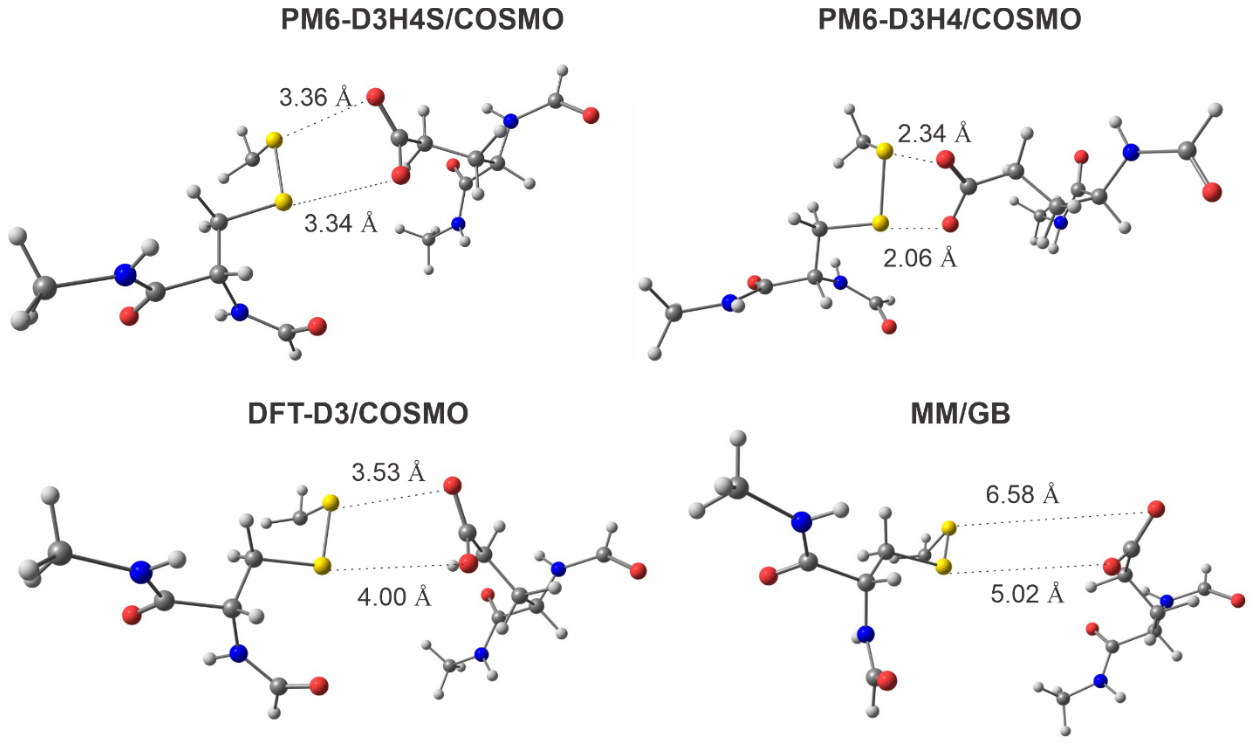

Fig S6. Charged chalcogen bonding at the CR/L2 domain interface of IR extracted from tr1/sn4. Structural description of interaction between disulfide bridge Cys 312…Cys 333 (left) and Glu 287 (right) as optimized by four different methods. For clarity, only S-CH_2 group of Cys 312 residue is shown. The distances for S…O chalcogen bonds are shown for the optimization protocols: PM6-D3H4S/COSMO (including the correction for S…O short-range repulsion), PM6-D3H4/COSMO (without the S…O correction), DFT-D3/COSMO and MM/GB.

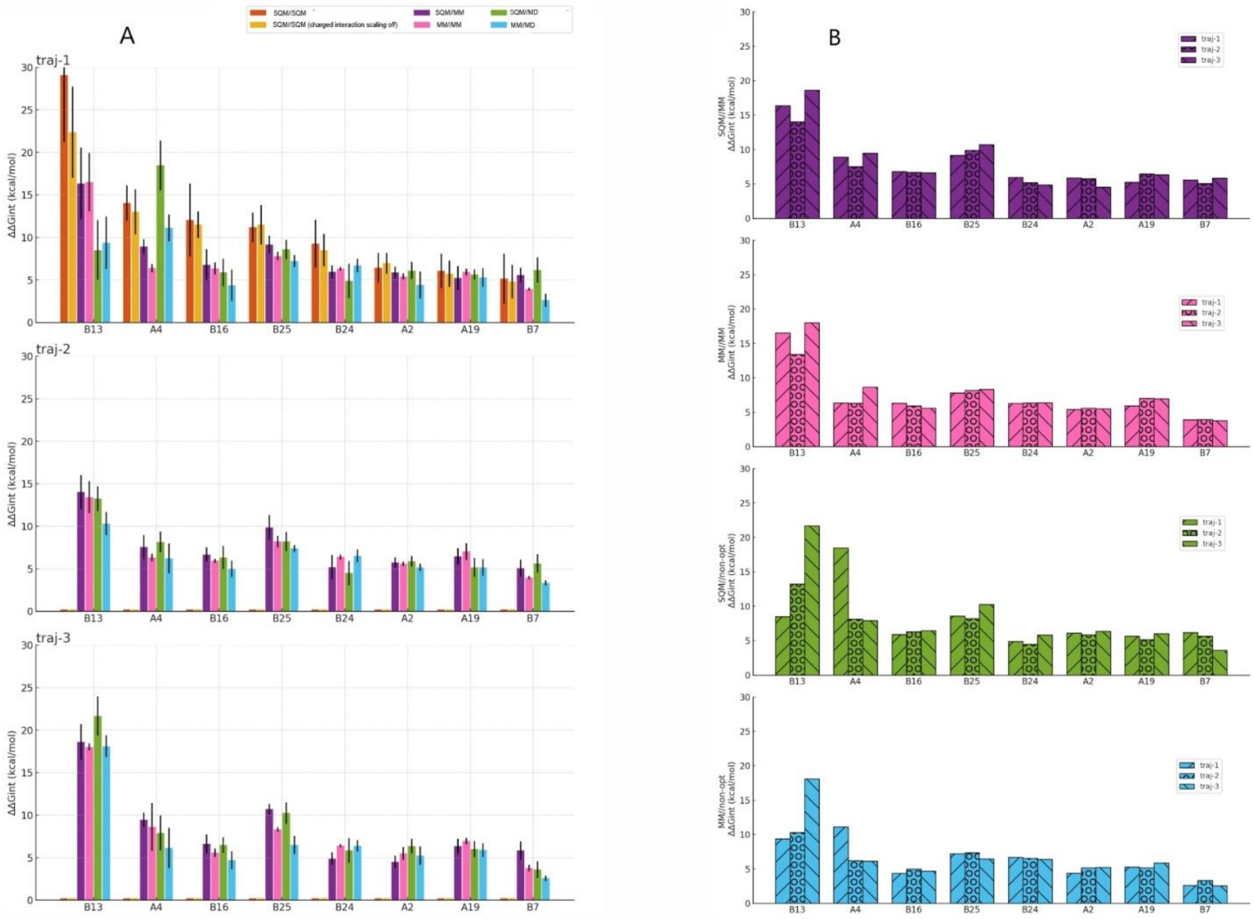

Fig. S7. **A.** The VGS energy contributions $\Delta\Delta G_{int}$ for eight selected residues (kcal/mol). **A.** Comparison of various computational protocols for ten snapshots of traj-1, **B.** Comparison of values for traj-1, traj-2 and traj-3.

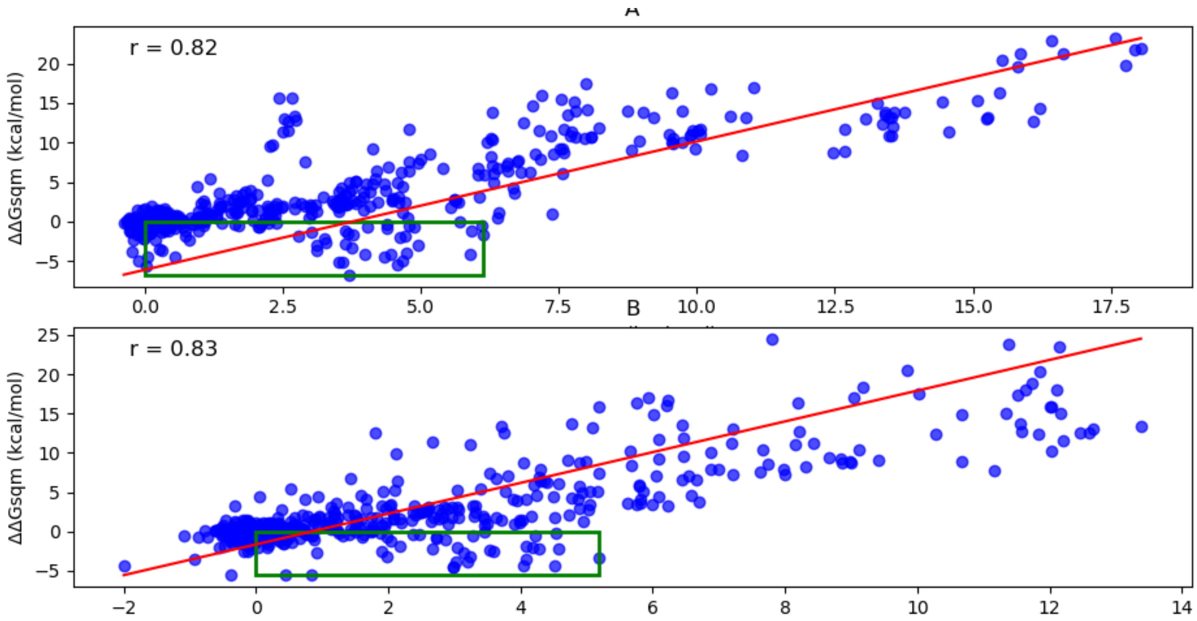

Fig. S8. Correlation in terms of Pearson's correlation coefficient, *r*, between SQM/COSMO2 and MM/GB insulin residue contributions in VGS obtained from all VGS runs performed for ten snapshots of the traj-1 for 11Å-models. **A.** MM/GB-optimized geometries, **B.** non-optimized MD geometries. The green rectangles indicate data points where the Gibbs free energy contributions were considered as destabilizing by PM6-D3H4S/COSMO2 but stabilizing by MM/GB.

Supplementary Tables

Table S1. Root-mean-square deviations (RMSD, in Å) of non-hydrogen atoms of 11 Å-fragmented complexes for the ten snapshots of traj-1. The non-optimized MD snapshots served as reference.

MD snapshot	Optimization method	
	MM/GB	PM6-D3H4S/COSMO
tr1-1	0.48	1.25
tr1-2	0.52	0.99
tr1-3	0.61	1.28
tr1-4	0.62	1.42
tr1-5	0.53	1.70
tr1-6	0.56	1.36
tr1-7	0.49	1.14
tr1-8	0.52	1.13
tr1-9	0.54	1.45
tr1-10	0.64	1.11

Table S2. Solvent-accessible surface area (SASA, 10^3 Å^2) computed for three sets of geometries (non-optimized MD, MM/GB and PM6-D3H4S/COSMO-optimized snapshots) for 10 selected 11 Å-fragmented snapshots from traj-1. The distance (in Å) between insulin Phe B1 and Lys B29 C_α atoms is shown as an additional measure of insulin molecule compactness within the insulin–IR optimized fragments.

	Solvent-accessible surface area (10^3 Å^2)						Distance (Å)		
	Insulin–IR fragment			Insulin			Insulin		
Geometries	MD	MM/GB	SQM/COSMO	MD	MM/GB	SQM/COSMO	MD	MM/GB	SQM/COSMO
tr1-1	24.6	24.7	22.7	6.1	6.2	5.8	35.0	34.1	31.9
tr1-2	25.0	25.0	23.1	6.1	6.1	5.8	33.6	34.7	31.2
tr1-3	25.2	25.3	23.4	6.1	6.1	5.8	33.2	33.5	32.3
tr1-4	26.5	26.5	24.1	6.1	6.2	5.8	33.0	35.0	30.6
tr1-5	26.3	26.4	24.4	6.1	6.2	5.8	33.8	34.4	31.4
tr1-6	25.3	25.5	23.5	6.1	6.1	5.9	33.7	34.3	32.7
tr1-7	25.3	25.5	22.8	6.1	6.2	5.7	34.4	35.2	28.3
tr1-8	25.5	25.7	23.5	6.1	6.1	5.8	34.6	35.1	33.8
tr1-9	24.6	24.7	22.6	6.1	6.1	5.9	34.4	34.6	32.3
tr1-10	25.4	25.5	23.5	6.1	6.2	5.8	34.6	34.8	33.2

Table S3. The interaction energies (kcal/mol) between insulin and IR calculated for 10 snapshots of each traj-1, traj-2 and traj-3 using various optimization protocols. For each MD snapshot, it is listed from which part of the trajectory it comes.

			Geometry				
Snapshot	MD Time (ns)		Non-optimized MD		MM/GB	PM6-D3H4S/COSMO	
			Energy				
			$\Delta G_{MM/GB}$	$\Delta G_{SQM/COSMO2}$	$\Delta G_{MM/GB}$	$\Delta G_{SQM/COSMO2}$	$\Delta G_{SQM/COSMO2}$
tr1-1	0.56	traj-1	-99.86	-65.14	-151.15	-83.21	-251.99
tr1-2	1.07		-101.23	-53.66	-151.86	-71.28	-237.23
tr1-3	1.14		-100.29	-60.96	-163.61	-92.02	-260.54
tr1-4	2.55		-106.96	-66.13	-154.40	-83.58	-265.20
tr1-5	3.00		-106.29	-53.76	-159.21	-92.64	-254.25
tr1-6	4.26		-106.64	-56.51	-151.59	-81.72	-275.26
tr1-7	4.73		-103.36	-61.00	-150.89	-77.59	-268.28
tr1-8	4.76		-99.71	-58.06	-151.87	-97.84	-271.69
tr1-9	4.85		-106.41	-62.29	-151.77	-92.63	-279.26
tr1-10	4.97		-103.63	-61.11	-146.49	-73.69	-237.79
tr2-1	2.33	traj-2	-97.54	-72.41	-134.07	-73.02	-
tr2-2	2.81		-98.01	-58.48	-140.57	-64.58	-
tr2-3	3.86		-99.19	-52.85	-139.59	-75.40	-
tr2-4	4.19		-102.14	-74.31	-140.42	-76.43	-
tr2-5	4.48		-99.78	-53.51	-141.71	-74.93	-
tr2-6	4.67		-97.72	-62.81	-135.58	-62.81	-
tr2-7	4.71		-102.35	-65.15	-136.46	-66.71	-
tr2-8	4.86		-104.66	-71.37	-145.25	-82.82	-
tr2-9	4.95		-104.44	-73.34	-153.05	-70.63	-
tr2-10	5.00		-102.90	-60.18	-147.94	-72.14	-
tr3-1	0.11	traj-3	-93.28	-73.02	-135.27	-78.02	-
tr3-2	0.21		-96.06	-72.21	-133.74	-74.58	-
tr3-3	0.26		-90.94	-50.00	-133.70	-85.27	-
tr3-4	3.47		-93.25	-77.42	-129.13	-82.05	-
tr3-5	3.58		-96.44	-63.93	-128.92	-68.60	-
tr3-6	3.74		-92.27	-65.41	-133.56	-87.83	-
tr3-7	3.89		-90.75	-64.33	-136.83	-83.53	-
tr3-8	4.00		-94.95	-70.14	-131.46	-83.33	-
tr3-9	4.05		-91.56	-68.32	-136.20	-75.55	-
tr3-10	4.21		-93.28	-78.62	-128.79	-79.04	-

Table S4. Averaged Interaction Energies (kcal/mol) for Characteristic Insulin–IR Dimers Computed Using DFT-D3/COSMO, PM6-D3H4S/COSMO2, and MM/GB Methods. The initial geometries of dimers were extracted from three snapshots: traj-1/sn-4, traj-2/sn-7, and traj-3/sn-5. The dimers were optimized at the DFT-D3(BJ) level of theory using the BLYP functional and def2-TZVPPD basis set with the COSMO implicit solvation model for water. Interaction energies are reported as average ± standard deviation across the three snapshots.

Insulin or IR residue pair	Interaction type	DFT-D3 /COSMO (kcal/mol)	PM6-D3H4S /COSMO2 (kcal/mol)	MM/GB (kcal/mol)
Glu A4 – Asn 711 (αCT')	NH…O	-17.87 ± 1.96	-17.00 ± 3.35	-12.80 ± 2.18
His B10 – Ser 827 (FnIII-1')	NH…O	-11.80 ± 3.82	-12.07 ± 5.47	-8.40 ± 1.45
Glu B13 – Arg 65 (L1)	NH…O, salt bridge	-93.07 ± 12.17	-91.00 ± 16.13	-79.27 ± 9.94
Glu B13 – Arg 539 (FnIII-1')	NH…O, salt bridge	-98.17 ± 15.27	-99.03 ± 18.75	-86.20 ± 9.38
Glu B13 – Ser 540 (FnIII-1')	OH…O	-22.90 ± 0.69	-23.73 ± 2.99	-21.60 ± 2.26
Leu B15 – Phe 714 (FnIII-1')	CH…π	-4.40 ± 2.92	-3.63 ± 2.44	-3.40 ± 1.85
Tyr B16 – Phe 39 (L1)	Stacking	-5.67 ± 0.80	-5.34 ± 0.71	-4.60 ± 0.97
Phe B24 – Asn 15 (L1)	NH…O	-6.67 ± 1.28	-6.67 ± 1.35	-5.20 ± 0.71
Phe B25 – Arg 14 (L1)	NH…O, charged	-20.23 ± 2.87	-20.30 ± 3.69	-16.07 ± 2.33
Glu 287 – Cys 333 (L2)	S…O, charged	-2.45 ± 1.45	-1.65 ± 0.84	+5.67 ± 0.66
Gly 463 – Cys 468 (L2)	S…O	-11.07 ± 1.21	-11.97 ± 1.50	-11.17 ± 2.07

Table S5. Percentage P of Site 1b VGS contribution to the total interaction energy ΔG_{int} calculated at the PM6-D3H4S/COSMO2 level for MM/GB optimized geometries. Minimal (P_{min}), maximal (P_{max}), and average ($P_{average}$) values are shown.

Model	ΔG_{int} (average), kcal/mol	P_{min} (Site 1b), %	P_{max} (Site 1b), %	$P_{average}$ (Site 1b), %
traj-1	-121.8	15.4	36.1	26.7
traj-2	-104.6	19.7	27.5	24.0
traj-3	-103.7	21.8	29.0	25.4

Table S6. The VGS free energy contributions, $\Delta\Delta G_{int}$ (kcal/mol) with RMSE values in parentheses for various computational protocols averaged over 10 selected snapshots of each trajectory. SQM//SQM stands for PM6-D3H4S/COSMO2// PM6-D3H4S/COSMO. The values are sorted by descending order of contributions in each protocol and trajectory.

Trajectory	Residue	SQM//SQM	SQM//SQM (charged interaction scaling off)	SQM//MM	MM//MM	SQM//MD	MM//MD
traj-1	B13	29.09 (7.87)	22.36 (5.36)	16.34 (4.22)	16.51 (3.43)	8.50 (3.51)	9.38 (3.09)
	B9	18.20 (6.14)	19.70 (5.13)	6.41 (2.82)	6.45 (1.35)	1.96 (0.28)	1.74 (0.21)
	B29	17.93 (8.34)	12.45 (6.81)	1.97 (0.34)	1.72 (0.25)	1.39 (0.34)	1.45 (0.42)
	B3	14.83 (4.85)	8.77 (2.09)	1.72 (0.12)	1.49 (0.29)	0.96 (0.18)	1.01 (0.12)
	A4	14.03 (2.06)	13.02 (2.65)	8.93 (0.85)	6.38 (0.50)	18.46 (2.94)	11.13 (1.59)
	B4	12.11 (4.78)	10.80 (3.70)	1.20 (0.17)	1.62 (0.30)	0.73 (0.19)	0.87 (0.23)
	B16	12.04 (4.29)	11.50 (1.56)	6.78 (1.84)	6.35 (0.71)	5.89 (1.61)	4.37 (1.84)
	B25	11.19 (1.73)	11.49 (2.32)	9.15 (1.04)	7.81 (0.49)	8.60 (1.13)	7.23 (0.72)
	B10	10.40 (2.93)	12.36 (1.75)	7.07 (2.55)	6.74 (0.62)	5.88 (2.08)	4.90 (3.02)
	B12	10.15 (3.05)	10.05 (2.01)	8.36 (1.78)	7.51 (0.52)	1.92 (0.45)	1.81 (0.34)
	B24	9.28 (2.82)	8.50 (1.91)	5.94 (0.77)	6.31 (0.23)	4.88 (2.03)	6.69 (0.81)
	B21	7.23 (2.60)	3.07 (2.36)	5.28 (1.63)	5.25 (0.46)	1.12 (0.10)	1.16 (0.17)
	A3	6.48 (2.23)	6.61 (0.91)	1.14 (0.12)	1.32 (0.21)	0.56 (0.08)	0.43 (0.10)
	A2	6.43 (1.73)	6.96 (1.22)	5.87 (0.68)	5.41 (0.35)	6.10 (1.01)	4.41 (1.57)
	A19	6.05 (2.03)	5.74 (1.55)	5.23 (1.41)	5.93 (0.40)	5.64 (0.62)	5.28 (1.10)
	B7	5.15 (2.93)	4.80 (1.97)	5.57 (0.84)	3.92 (0.16)	6.16 (1.50)	2.61 (0.77)
	B15	4.42 (1.17)	4.93 (0.67)	3.49 (0.46)	2.91 (0.10)	2.97 (0.48)	2.15 (0.90)
traj-2	B25	-	-	9.87 (1.45)	8.20 (0.69)	8.22 (1.14)	7.39 (0.40)
	B12	-	-	8.33 (1.56)	7.04 (0.40)	8.26 (1.73)	5.10 (0.88)
	A4	-	-	7.55 (1.41)	6.32 (0.45)	8.15 (1.22)	6.23 (1.75)
	B16	-	-	6.67 (0.84)	5.93 (0.25)	6.31 (1.37)	4.98 (0.95)
	A19	-	-	6.46 (0.95)	7.04 (0.98)	5.17 (1.12)	5.17 (0.97)
	A2	-	-	5.75 (0.60)	5.59 (0.27)	5.88 (0.62)	5.17 (0.44)
	B24	-	-	5.19 (1.44)	6.38 (0.28)	4.49 (1.42)	6.54 (0.75)
	B7	-	-	5.08 (1.00)	3.97 (0.18)	5.65 (1.09)	3.34 (0.29)
traj-3	B13	-	-	18.61 (2.08)	18.02 (0.41)	21.67 (2.30)	18.10 (1.29)
	B25	-	-	10.70 (0.63)	8.33 (0.27)	10.26 (1.25)	6.49 (1.07)
	B12	-	-	9.88 (1.33)	7.31 (0.62)	7.25 (2.01)	5.20 (0.83)
	A4	-	-	9.46 (0.83)	8.62 (2.81)	7.90 (2.07)	6.14 (2.38)
	B16	-	-	6.62 (1.10)	5.59 (0.49)	6.48 (0.91)	4.70 (1.05)
	B4	-	-	6.50 (0.94)	6.31 (0.60)	3.94 (1.28)	4.33 (1.04)
	A19	-	-	6.35 (0.88)	6.97 (0.36)	6.01 (0.97)	5.90 (0.80)
	B7	-	-	5.83 (1.07)	3.79 (0.37)	1.92 (0.34)	1.74 (0.21)
	B24	-	-	4.88 (0.76)	6.42 (0.19)	5.84 (1.44)	6.40 (0.68)
	A2	-	-	4.51 (0.71)	5.50 (0.76)	6.36 (0.86)	5.24 (1.09)
	A3	-	-	4.14 (0.71)	8.70 (0.36)	1.94 (0.49)	1.99 (0.52)
	B15	-	-	2.68 (0.36)	2.81 (0.08)	2.72 (0.44)	2.12 (0.96)

Table S7. Insulin hotspots residues obtained from VGS on snapshots from three MD trajectories, each optimized using MM/GB approach. $\Delta\Delta G_{int}$ values at the PM6-D3H4S/COSMO2 level are listed in descending order. Means over 10 snapshots and standard deviations are listed. All the values are in kcal/mol.

Trajectory	Insulin hotspots residues	$\Delta\Delta G_{int}$ (PM6-D3H4S/ COSMO2)	$\Delta\Delta E_{int}$ (MM/GB)	Interactions with IR
traj-1	GLU B13[a,b]	16.34 (4.22)	16.51 (3.43)	ARG 65 (L1), ARG 539, SER 540 (both FnIII-1')
	PHE B25 [a]	9.15 (1.04)	7.81 (0.49)	ARG 14 (L1), ARG 717 (αCT')
	GLU A4 [a]	8.93 (0.85)	6.38 (0.50)	ASN 711 (αCT')
	VAL B12 [a]	8.36 (1.78)	7.51 (0.52)	PHE 39, ARG 65 (both L1),
	HIS B10 [b]	7.07 (2.55)	6.74 (0.62)	SER 540 (FnIII-1')
	TYR B16 [a]	6.78 (1.84)	6.35 (0.71)	PHE 39 (L1)
	SER B9 [a]	6.41 (2.82)	6.45 (1.35)	ARG 65 (L1)
	PHE B24 [a]	5.94 (0.77)	6.31 (0.23)	ASN 15 (L1), LEU 37 (L1), PHE 714 (αCT')
	ILE A2 [a]	5.87 (0.68)	5.41 (0.35)	ASN 711, PHE 714 (both αCT')
	CYS B7[b]	5.57 (0.84)	3.92 (0.16)	ASP 496, ARG 498 (both FnIII-1')
	GLU B21 [a]	5.28 (1.63)	5.25 (0.46)	LYS 40 (L1)
	TYR A19 [a]	5.23 (1.41)	5.93 (0.40)	VAL 713 (αCT')
	LEU B15 [a]	3.49 (0.46)	2.91 (0.10)	PHE 714 (αCT')
traj-2	GLU B13 [a,b]	14.03 (2.02)	13.44 (1.89)	SER 540 (FnIII-1'), ARG 65 (L1)
	PHE B25 [a]	9.87 (1.45)	8.20 (0.69)	ARG 14 (L1)
	VAL B12 [a]	8.33 (1.56)	7.04 (0.40)	HIE 710 (αCT')
	GLU A4 [a]	7.55 (1.41)	6.32 (0.45)	ASN 711 (αCT')
	TYR B16 [a]	6.67 (0.84)	5.93 (0.25)	PHE 39 (L1)
	TYR A19 [a]	6.46 (0.95)	7.04 (0.98)	PHE 714, PRO 716 (both αCT')
	ILE A2 [a]	5.75 (0.60)	5.59 (0.27)	PHE 714 (αCT')
	PHE B24 [a]	5.19 (1.44)	6.38 (0.28)	ASN 15, LEU 37 (both L1)
	CYS B7 [b]	5.08 (1.00)	3.97 (0.18)	ASP 496, ARG 498 (both FnIII-1')
traj-3	GLU B13 [a,b]	18.61 (2.08)	18.02 (0.41)	ARG 65 (L1), ARG 539 (FnIII-1')
	PHE B25 [a]	10.70 (0.63)	8.33 (0.27)	ARG 14 (L1), VAL 715, ARG 717 (both αCT')
	VAL B12 [a]	9.88 (1.33)	7.31 (0.62)	PHE 39 (L1) and PHE 714 (αCT')
	GLU A4 [a]	9.46 (0.83)	8.62 (2.81)	ASN 711 (αCT')
	TYR B16 [a]	6.62 (1.10)	5.59 (0.49)	PHE 39 (L1)
	GLN B4 [b]	6.50 (0.94)	6.31 (0.60)	SER 540, TRP 493 (both FnIII-1')
	TYR A19 [a]	6.35 (0.88)	6.97 (0.36)	PRO 716, VAL 715 (both αCT')
	CYS B7 [b]	5.83 (1.07)	3.79 (0.37)	ASP 496, ARG 498 (both FnIII-1')
	PHE B24 [a]	4.88 (0.76)	6.42 (0.19)	ASN 15, LEU 37 (both L1)
	ILE A2 [a]	4.51 (0.71)	5.50 (0.76)	PHE 714, HIE 710 (both αCT')
	VAL A3 [a]	4.14 (0.71)	8.70 (0.36)	ASN 711 (αCT')
	LEU B15 [a]	2.68 (0.36)	2.81 (0.08)	PHE 714 (αCT')

Table S8. IR hotspots residues obtained from VGS on snapshots from three MD trajectories, each optimized using MM/GB approach. $\Delta\Delta G_{int}$ values at the PM6-D3H4S/COSMO2 level are listed in descending order. Means over 10 snapshots and standard deviations are listed. All the values are in kcal/mol.

Trajectory	IR hotspot residues	$\Delta\Delta G_{int}$ (PM6-D3H4S/ COSMO2)	$\Delta\Delta E_{int}$ (MM/GB)	Interactions with insulin
traj-1	ARG 65 (L1)	20.85 (1.96)	16.71 (1.04)	B9 (SER), B13 (GLU), B12 (VAL)
	SER 540 (FnIII-1’)	14.33 (1.73)	9.66 (1.07)	B13 (GLU), B10 (HIS)
	ASN 711 (αCT’)	13.38 (0.83)	13.44 (0.20)	A4 (GLU), A2 (ILE)
	ARG 498 (FnIII-1’)	12.74 (1.90)	7.62 (0.52)	B7 (CYS)
	ASP 12 (L1)	12.54 (2.07)	2.53 (0.16)	B26 (TYR)
	ARG 717 (αCT’)	12.15 (2.35)	14.19 (1.42)	A18 (ASN), A20 (CYX), A21 (ASN), B25 (PHE)
	PHE 714 (αCT’)	10.92 (0.59)	9.82 (0.22)	B15 (LEU), A2 (ILE), B24 (PHE)
	ARG 14 (L1)	10.32 (1.59)	7.14 (0.82)	B25 (PHE)
	PHE 39 (L1)	6.82 (0.82)	6.56 (0.34)	B16 (TYR), B12 (VAL)
	ASP 496 (FnIII-1’)	5.48 (2.09)	4.42 (0.58)	B7 (CYX)
	TRP 493 (FnIII-1’)	2.85 (0.80)	3.90 (0.29)	B4 (GLN)
traj-2	ARG 65 (L1)	17.57 (2.25)	14.36 (0.62)	B13 (GLU)
	ARG 498 (FnIII-1’)	14.61 (2.06)	8.34 (0.43)	A7 (CYX), B7 (CYX), B8 (GLY)
	ASP 12 (L1)	13.01 (1.88)	2.52 (0.28)	B26 (TYR)
	ASN 711 (αCT’)	12.54 (0.77)	13.47 (0.56)	A3 (VAL), A4 (GLU)
	PHE 714 (αCT’)	11.01 (0.72)	9.81 (0.31)	B24 (PHE), A2 (ILE), B15 (LEU)
	ARG 14 (L1)	8.32 (2.12)	7.07 (0.51)	B25 (PHE)
	SER 540 (FnIII-1’)	8.28 (1.50)	7.78 (0.77)	B13 (GLU), B5 (HIS)
	PHE 39 (L1)	6.61 (0.76)	6.41 (0.20)	B16 (TYR)
	ARG 717 (αCT’)	6.09 (0.96)	10.69 (1.00)	A18 (ASN), B25 (PHE)
	ASP 496 (FnIII-1’)	5.63 (0.93)	5.06 (0.36)	B7 (CYS)
traj-3	ARG 65 (L1)	20.78 (0.86)	17.46 (0.44)	B13 (GLU), B9 (SER)
	ARG 498 (FnIII-1’)	15.76 (1.93)	8.34 (0.53)	A7 (CYS), B7 (CYS), B8 (GLY)
	ASP 12 (L1)	13.30 (1.51)	2.81 (0.18)	B26 (TYR)
	ASN 711 (αCT’)	12.69 (1.72)	13.70 (0.42)	A3 (VAL), A4 (GLU)
	ARG 14 (L1)	10.19 (1.92)	7.90 (0.45)	B25 (PHE)
	ARG 539 (FnIII-1’)	9.83 (1.57)	12.67 (0.21)	B13 (GLU)
	PHE 714 (αCT’)	9.57 (0.76)	9.23 (0.27)	B24 (PHE), B15 (LEU)
	GLU 706 (αCT’)	6.85 (2.99)	5.40 (0.92)	A2 (ILE), A4 (GLU)
	PHE 39 (L1)	6.44 (0.57)	6.50 (0.34)	B16 (TYR)
	ASN 15 (L1)	6.00 (0.95)	4.47 (0.10)	B24 (PHE)

Table S9. Insulin hotspots residues obtained from VGS on snapshots from three MD trajectories, without optimization. $\Delta\Delta G_{int}$ values at the PM6-D3H4S/COSMO2 level are listed in descending order. Means over 10 snapshots and standard deviations are listed. All the values are in kcal/mol.

Trajectory	Insulin hotspots residues	$\Delta\Delta G_{int}$ (PM6-D3H4S/COSMO2)	$\Delta\Delta E_{int}$ (MM/GB)	Interactions with IR
traj-1	GLU A4[a]	18.46 (2.94)	11.13 (1.59)	ASN 711 (αCT')
	PHE B25[a]	8.60 (1.13)	7.23 (0.72)	ARG 14 (L1)
	GLU B13[b]	8.50 (3.51)	9.38 (3.09)	SER 540 (FnIII-1')
	CYS B7[a]	6.16 (1.50)	2.61 (0.77)	ASP 496 (FnIII-1'), ARG 498 (FnIII-1')
	ILE A2[a]	6.10 (1.01)	4.41 (1.57)	PHE 714 (αCT')
	TYR B16[a]	5.89 (1.61)	4.37 (1.84)	PHE 39 (L1)
	HIS B10[b]	5.88 (2.08)	4.90 (3.02)	SER 540 (FnIII-1'), PHE 497 (FnIII-1')
	TYR A19[a]	5.64 (0.62)	5.28 (1.10)	PHE 714 (αCT')
	PHE B24[a]	4.88 (2.03)	6.69 (0.81)	ASN 15 (L1), LEU 37 (L1), PHE 714 (αCT')
	LEU B15[a]	2.97 (0.48)	2.15 (0.90)	PHE 714 (αCT')
traj-2	GLU B13[a]	13.24 (1.46)	10.32 (1.37)	ARG 65 (L1)
	VAL B12[a]	8.26 (1.73)	5.10 (0.88)	PHE 39, PHE 64 (both L1), HIE 710, PHE 714 (both αCT')
	PHE B25[a]	8.22 (1.14)	7.39 (0.40)	ARG 14 (L1)
	GLU A4[a]	8.15 (1.22)	6.23 (1.75)	ASN 711 (αCT')
	TYR B16[a]	6.31 (1.37)	4.98 (0.95)	PHE 39 (L1)
	ILE A2 [a]	5.88 (0.62)	5.17 (0.44)	HIE 710, PHE 714 (both αCT')
	CYS B7[b]	5.65 (1.09)	3.34 (0.29)	ARG 498 (FnIII-1')
	TYR A19[a]	5.17 (1.12)	5.17 (0.97)	PHE 714, PRO 716 (both αCT')
	PHE B24[a]	4.49 (1.42)	6.54 (0.75)	ASN 15 (L1), PHE 714 (αCT')
	VAL A3[a]	3.85 (0.98)	6.22 (0.87)	ASP 707, ASN 711 (both αCT')
	LEU B15[a]	3.33 (0.57)	2.57 (0.39)	PHE 714 (αCT')
traj-3	GLU B13[a,b]	21.67 (2.30)	18.10 (1.29)	ARG 539 (FnIII-1'), ARG 65 (L1)
	PHE B25[a]	10.26 (1.25)	6.49 (1.07)	ARG 14 (L1), VAL 715, ARG 717 (both αCT')
	GLU A4 [a]	7.90 (2.07)	6.14 (2.38)	ASN 711 (αCT')
	VAL B12 [a]	7.25 (2.01)	5.20 (0.83)	PHE 39 (L1), PHE 714 (αCT')
	TYR B16 [a]	6.48 (0.91)	4.70 (1.05)	PHE 39 (L1)
	ILE A2 [a]	6.36 (0.86)	5.24 (1.09)	HIE 710 (αCT'), PHE 714 (αCT')
	TYR A19 [a]	6.01 (0.97)	5.90 (0.80)	VAL 715 (αCT'), PRO 716 (αCT')
	PHE B24 [a]	5.84 (1.44)	6.40 (0.68)	ASN 15 (L1), PHE 39 (L1)
	GLN B4[b]	3.94 (1.28)	4.33 (1.04)	SER 540 (FnIII-1'), TRP 493 (FnIII-1')
	LEU B15[a]	2.72 (0.44)	2.12 (0.96)	PHE 714 (αCT')

Table S10. IR hotspots residues obtained from VGS on snapshots from three MD trajectories, without optimization. $\Delta\Delta G_{int}$ values at the PM6-D3H4S/COSMO2 level are listed in descending order. Means over 10 snapshots and standard deviations are listed. All the values are in kcal/mol.

Trajectory	IR hotspots residues	$\Delta\Delta G_{int}$ (PM6-D3H4S/ COSMO2)	$\Delta\Delta E_{int}$ (MM/GB)	Interactions with insulin
traj-1	ARG 65 (L1)	18.46 (2.94)	11.13 (1.59)	B9 (SER), B13 (GLU)
	ASN 711 (αCT’)	17.61 (3.70)	10.55 (1.62)	A4 (GLU), A2 (ILE)
	SER 540 (FnIII-1’)	14.56 (3.64)	5.80 (2.44)	B13 (GLU)
	ARG 498 (FnIII-1’)	13.20 (1.95)	5.60 (0.85)	B7 (CYS)
	ARG 717 (αCT’)	10.41 (1.84)	11.82 (0.94)	A21 (ASN), A19 (TYR)
	ARG 14 (L1)	9.63 (2.54)	7.17 (1.08)	B25 (PHE), B26 (TYR)
	PHE 714 (αCT’)	8.61 (1.17)	8.30 (0.88)	B15 (LEU), B24 (PHE)
	ASP 496 (FnIII-1’)	7.60 (1.90)	4.84 (0.81)	B7 (CYS)
	ASP 12 (L1)	6.13 (3.35)	1.81 (0.46)	B26 (TYR)
	ASN 15 (L1)	6.04 (0.97)	3.93 (0.56)	B24 (PHE)
	PHE 39 (L1)	5.02 (1.66)	5.96 (0.43)	B16 (TYR), B12 (VAL), B24 (PHE)
traj-2	ARG 65 (L1)	19.44 (2.38)	11.50 (1.42)	B13 (GLU)
	ASN 711 (αCT’)	16.18 (3.26)	10.75 (1.35)	A3 (VAL), A4 (GLU)
	ARG 785 (FnIII-1’)	14.19 (2.67)	6.36 (0.39)	A7 (CYS), B7 (CYS), B8 (GLY)
	SER 540 (FnIII-1’)	9.92 (1.54)	5.30 (1.14)	B13 (GLU)
	PHE 714 (αCT’)	8.92 (1.75)	8.50 (0.85)	B15 (LEU), B24 (PHE)
	ARG 14 (L1)	7.84 (1.47)	6.82 (0.64)	B25 (PHE)
	ASP 496 (FnIII-1’)	7.59 (2.41)	5.28 (0.74)	B7 (CYS)
	ASP 12 (L1)	6.38 (2.71)	1.59 (0.44)	B26 (TYR)
	PHE 39 (L1)	5.44 (0.77)	5.55 (1.10)	B16 (TYR)
traj-3	ARG 65 (L1)	21.12 (2.14)	15.74 (1.97)	B9 (SER), B13 (GLU)
	ARG 498 (FnIII-1’)	16.73 (2.44)	6.61 (0.89)	A7 (CYS), B7 (CYS), B8 (GLY)
	ASN 711 (αCT’)	16.54 (3.42)	11.03 (2.52)	A3 (VAL), A4 (GLU)
	ARG 539 (FnIII-1’)	11.68 (1.09)	12.22 (0.57)	B13 (GLU)
	ARG 14 (L1)	9.86 (1.58)	6.97 (1.04)	B25 (PHE)
	PHE 714 (αCT’)	8.32 (1.40)	7.70 (1.78)	B24 (PHE)
	ASP 12 (L1)	7.57 (2.22)	1.93 (0.52)	B26 (TYR)
	GLU 706 (αCT’)	7.10 (1.83)	4.55 (1.07)	A2 (ILE), A4 (GLU)
	ASN 15 (L1)	6.73 (1.68)	3.18 (0.80)	B24 (PHE)
	PHE 39 (L1)	5.39 (0.93)	5.31 (1.33)	B16 (TYR)

Table S11. Performance of various combinations of computational protocols (single-point energy//geometry optimization) in detection of interaction hotspots, time requirement for individual optimization and single-point steps. The data are shown based on analysis of 11Å-fragmented snapshots from traj-1.

Computational protocol	Number of interaction hotspots identified	List of missed hotspots with respect to SQM//SQM protocol	Timing (in hours) for a single 11Å-fragmented complex, 1 CPU	
			Geometry optimization	Single point energy calculation
SQM//SQM	15	-	up to ~250	~0.3-0.6
SQM//MM	15	-	3-4	~0.3-0.6
MM//MM	15	-	3-4	~0.006
SQM//MD	13	Asn B3, Gln B4	0	~0.3-0.6
MM//MD	12	Asn B3, Gln B4, Leu B15	0	~0.006

Table S12. Comparison of VGS data with biochemical experiments (alanine scanning mutagenesis).[2] Relative affinities of insulin single-point alanine mutants represent the percentages affinities with respect to wild-type insulin.

Site	Residue	Residue location	Soluble receptor Rel.affinity of Ala mutant	Cell receptor Rel.affinity of Ala mutant
1	Ile A2	N-terminal α-helix	-	0.6
	Glu A4	N-terminal α-helix	139	142
	Tyr A19	C-terminal segment	0	<0.1
	Val B12	Central α-helix	-	1
	Tyr B16	Central α-helix	69	34
	Phe B24	C-terminal β-strand	-	5
	Phe B25	C-terminal β-strand	-	10
2	Cys B7	type II β-turn	-	-
	Glu B13	Central α-helix	12	10

References

1. R. Johnson, S. Keinan, P. Mori-Sanchez, J. Contreras-Garcia, A. J. Cohen, W. Yang. Revealing Noncovalent Interactions. *J. Am. Chem. Soc.*, 2010, 132, 6498
2. P. De Meyts. Insulin/receptor binding: The last piece of the puzzle? What recent progress on the structure of the insulin/receptor complex tells us (or not) about negative cooperativity and activation. *BioEssays*, 2015, 37, 389
